# Intratumoral microenvironment remodeling by lncRNA ROLLCSC enhances lung adenocarcinoma progression

**DOI:** 10.1016/j.gendis.2025.101788

**Published:** 2025-08-05

**Authors:** Yu-Han Zhang, Jia-Cheng Xie, Ting Ye, Shi-Meng Guo, Xue Han, Si Yang, Lei Shi, Yi-Shi Li, H. Rosie Xing, Jing-Yu Li, Jian-Yu Wang

**Affiliations:** a Chongqing Key Laboratory of Human Embryo Engineering and Precision Medicine, NHC Key Laboratory of Birth Defects and Reproductive Health, Center for Reproductive Medicine, Chongqing Health Center for Women and Children, Women and Children’s Hospital of Chongqing Medical University, Chongqing 400016, China; b Molecular Biology Laboratory of Respiratory Disease, Key Laboratory of Clinical Laboratory Diagnostics (Ministry of Education), College of Laboratory Medicine, Chongqing Medical University, Chongqing 400016, China; c State Key Laboratory of Ultrasound in Medicine and Engineering, College of Biomedical Engineering, Chongqing Medical University, Chongqing 400016, China; d Department of Laboratory Medicine, The Affiliated Hospital of Southwest Medical University, Sichuan 646000, China; e Department of Respiratory and Critical Care Medicine, The First Affiliated Hospital of Chongqing Medical University, Chongqing 400016, China

**Keywords:** Cancer stem cells, Extracellular vesicles, Ferreptosis, Lipid metabolism, Long-noncoding RNA

## Abstract

Metabolic reprogramming is one of the eight hallmarks of cancer, and in lung cancer, it is notably linked to ferroptosis-related lipid metabolism. Cancer stem cells, regarded as the initiating cells of cancer, can extensively influence the tumor microenvironment (TME). Nevertheless, their role in metabolic reprogramming within lung adenocarcinoma (LUAD) remains incompletely explored. In this study, through molecular biology experiments including RNA-seq, proteomics, RNA pulldown, and PCR, we discovered a novel and intricate mechanism by which the lncRNA ROLLCSC, derived from extracellular vesicles (EVs) of LUAD stem cells, regulates the tumor TME. Mechanistically, lncRNA ROLLCSC can interact with CDC42, a GTPase protein, mediating a positive feedback loop that promotes the entry of more EVs into recipient lung cancer cells (LLC). FTO-mediated m6A demethylation enhances the stability of ROLLCSC, which is recognized by the reader protein IGF2BP2 in recipient LLC cells. Most importantly, lncRNA ROLLCSC can reshape the lipid metabolism of LLC cells by targeting ACSL4 and *Slc25a11*, thereby enhancing their resistance to ferroptosis. Clinically, ROLLCSC and its targets are associated with distinct tumor expression patterns and have prognostic significance. Overall, our study elucidates how the lncRNA ROLLCSC derived from cancer stem cell (CSC)-derived EVs is efficiently transported to LUAD cells, subsequently reshaping the lipid metabolism of recipient cells and enhancing their resistance to ferroptosis.

## Introduction

Lung adenocarcinoma (LUAD) is a highly invasive with an overall survival rate of less than five years.^1^ LUAD is often diagnosed at advanced metastatic stage, leading to a poor prognosis.^2^ Tumor microenvironment (TME) reprogramming is closely linked to tumor metastasis and progression. Cancer stem cells (CSCs) are believed to drive tumor progression by altering the intercellular communication in the TME.^3^^,^^4^ Recent studies have shown that intercellular communication mediated by extracellular vesicles (EVs) within the TME is a key mechanism through which CSCs influence tumor initiation and development. EVs are lipid nanovesicles, approximately 30–150 nm in size, derived from multivesicular bodies formed via endocytosis. EVs facilitate cell-to-cell communication by carrying various biologically active molecules produced through intracellular homeostatic mechanisms.^5^^,^^6^ For instance, RNA, which functions as a critical intermediate, is often detected as EVs cargo and plays a role in regulating cancer cell phenotypes, thus contributing to tumor progression.^7^^,^^8^ Long non-coding RNAs (lncRNAs), which are over 200 bp in length and do not directly encode proteins, have increasingly been shown to influence tumor progression at both the transcriptional and post-transcriptional levels.^9^^,^^10^ In our prior investigation, we discovered that LUAD stem cells through the extracellular vesicle lncRNA ROLLCSC augmented cellular lipid metabolism and facilitated LUAD metastasis by targeting miR-217-3p/5623-3p.^11^ However, the mechanisms underlying the encapsulation of lncRNA ROLLCSC into EVs and its role in driving lipid metabolic reprogramming in recipient LUAD cells remain largely unexplored.

Metabolic reprogramming is one of the key hallmarks of tumorigenesis, wherein the metabolic network of tumor cells is reconfigured to support their growth, proliferation, and metastasis under the metabolic stress of the TME.^12^^,^^13^ Increasing evidence indicates that EVs, as critical mediators of information transfer within the TME, can deliver biologically active molecules such as RNA, proteins, and lipids to recipient cells, thereby promoting cancer progression by reprogramming the metabolism of both cancer cells and surrounding stromal cells.^14^ In the metabolic reprogramming of tumors, the alteration of fatty acids is particularly significant, with EVs playing a key role in this process.^15^^,^^16^ The oxidation of free fatty acids serves as a primary energy source to sustain the high energy demands of tumor cells.^17^ The type and saturation of these fatty acids are crucial for their structural stability and function. Saturated fatty acids are essential for maintaining membrane fluidity and selective transmembrane transport. Unsaturated fatty acids, especially polyunsaturated fatty acids (PUFAs), are highly susceptible to reactive oxygen species (ROS), leading to lipid peroxidation and increasing cellular sensitivity to ferroptosis.^18^, ^19^, ^20^

Ferroptosis is a nonapoptotic, iron-dependent form of programmed cell death. It is induced by lipid peroxidation of phospholipids containing polyunsaturated fatty acid chains (PL-PUFAs) under the influence of Fe^2+^ or lipoxygenase. This process is regulated by multiple metabolic pathways, including glucose, iron, lipid, and GSH metabolism.^21^ Among these factors, GSH depletion and lipid peroxidation are the most direct factors leading to ferroptosis.^22^ Long-chain acyl-CoA synthetase 4 (ACSL4) promotes the formation of PUFA-PLs, whose peroxidative nature compromises plasma membrane integrity, resulting in the rapid accumulation of reactive oxygen species (ROS).^23^^,^^24^ Conversely, PUFAs are primarily β-oxidized in the mitochondria, a process dependent on mitochondrial membrane transporters (OGC, SLC25A11) for shuttling GSH, thereby counteracting excess mitochondrial ROS and reducing susceptibility to ferroptosis.^25^ However, little is known about the mechanisms by which lncRNAs influence ferroptosis during LUAD development.

In this research, we revealed the mechanism through which EVs lncRNAs facilitated the transfer of “metastatic capability” between LUAD stem cells (LLC-SD) and LUAD non-stemness cells (LLC). Specifically, *Cdc42* promotes the encapsulation of ROLLCSC into EVs and subsequently entry into recipient LLC cells. ROLLCSC augments the transcriptional activation of *Cdc42* by transcription factor Myc, which further enhances the uptake of EVs by recipient LLC cells through a positive feedback loop. In recipient LLC cells, the FTO-mediated m6A modification of ROLLCSC is recognized by the m6A reader IGF2BP2, enhancing RNA stability. This stabilization makes ROLLCSC more effective in reducing the ferroptosis susceptibility of LUADs by inhibiting ACSL4 and activating *Slc25a11*, thereby promoting tumor progression.

## Methods

### Cell lines and cell culture

The LLC cell line (mouse lung adenocarcinoma) was purchased from the Chinese Academy of Sciences Cell Bank. The LLC-SD cell line is a type of lung cancer stem cell displaying asymmetrical division that is separated and purified from LLC. Our group previously confirmed that LLC-SD cell lines can be stably passaged and distinguished on the basis of the following characteristics: spheroid formation ability, cancer stem cell marker expression and subcutaneous tumor initiation efficiency.^26^^,^^27^ LLC cells were propagated in adhesive culture dishes with DMEM supplemented with 10% FBS and 1% penicillin‒streptomycin. LLC-SD cells were cultured in DMEM/F12 1:1 (Baselmedia, D211015, China) supplemented with 1% B27 (Baselmedia, M431018, China) and 1% Pen Strep (Gibco, 15140–122, America). The cell culture conditions were 37 °C with 5% CO_2._

### Cell transfection and lentiviral infection

siRNAs and miRNA mimics were purchased from GenePharma (A01001). The transfection reagent used was Lipofectamine™ 2000 (Thermo, 11668019), and the cells were transfected when the degree of cell polymerization reached 50%. The RNA expression of the transfection complex was subsequently detected after the cells were cocultured for at least 48 h, and the protein expression was detected after the cells were cocultured for at least 72 h.

The lentiviruses were obtained from Genechem (Shanghai). Logarithmically proliferating cells were seeded in 24-well plates at a density of 5 × 10^4^ cells per well. Subsequently, 5 μL of lentivirus was added to each well, and the cells were coincubated for at least 48 h. After three stable passages, the transfection efficiency was detected via PCR/WB.

### Quantitative RT‒PCR (qRT‒PCR) analysis

To quantify the expression levels of specific genes accurately, we employed quantitative real-time fluorescence PCR (qRT‒PCR). First, total RNA was isolated via the RNA-Easy Isolation Reagent (Vazyme, R701-01). The isolated RNA was subsequently converted into cDNA via the HiScript II 1st Strand cDNA Synthesis Kit (Vazyme, R211-01). We utilized Universal SYBR qPCR Master Mix (Vazyme, Q511-02) for PCR analysis. The primers were specifically designed via Primer3plus (https://www.primer3plus.com/) and synthesized by Sangon Biotech (Shanghai) (Table S1). The qRT‒PCR data were rigorously analyzed via the 2ˆ‒ΔΔCt method and statistical significance was determined via GraphPad 8.0 software.

### Protein extraction and Western blot analysis

To efficiently lyse 5 × 10^5^ cells, we added 100 μl of RIPA buffer (Solarbio, R10010) and chilled the mixture on ice for 20 min. Following lysis, the mixture was centrifuged at 12,000 × *g* for 15 min to separate the cellular supernatant. The supernatant was mixed with 5x loading buffer (Solarbio, P1040) and boiled for 10 min to denature the proteins. It is essential to maintain the protein loading amount within the optimal range of 30–50 μg. The denatured proteins were separated via SDS‒PAGE electrophoresis with an 8%–12% polyacrylamide gel. The isolated proteins were then transferred to a PVDF membrane (Millipore, 0.45 μm, IPVH00010) and blocked with NcmBlot blocking buffer (NCMbiotech, P30500) for 15 min. Finally, the samples were incubated with a specific primary antibody (Table S2) overnight at 4 °C, washed thoroughly with 1x TBST buffer (NCMbiotech, WB20500) after which they were incubated with a secondary antibody for 1 h. Finally, enhanced chemiluminescence (ECL) substrates were used for observation under UV light (NCMbiotech, P2300).

### Luciferase reporter assay

Mutated and wild-type plasmids were purchased from Tsingke Biotechnology Co., Ltd (Table S3). 293T cells were used as the transfection vector. When the degree of cell polymerization reached 50% in the 96-well plate, the plasmid was transfected with Lipofectamine™ 2000 (Thermo, 11668019). After 48 h, the supernatant was removed, and the subsequent experiments were performed according to the instructions of the Dual Luciferase Reporter Gene Assay Kit (Beyotime, RG027).

### MDA, GSH, and ROS experiments

Cellular MDA, GSH, and ROS levels were determined via the Lipid Peroxidation MDA Assay Kit (Biosharp, BL904A), GSH content Determination Kit (Biosharp, BL874B), and Reactive Oxygen Species Assay Kit (Biosharp, BL714A), respectively, and the manufacturer’s instructions were strictly followed throughout the experiment. Finally, the values were detected with a microplate reader.

Further information is provided in the Supplementary Materials.

## Results

### CDC42 facilitates the encapsulation of ROLLCSC into LLCSD extracellular vesicles

**Figure 1:**
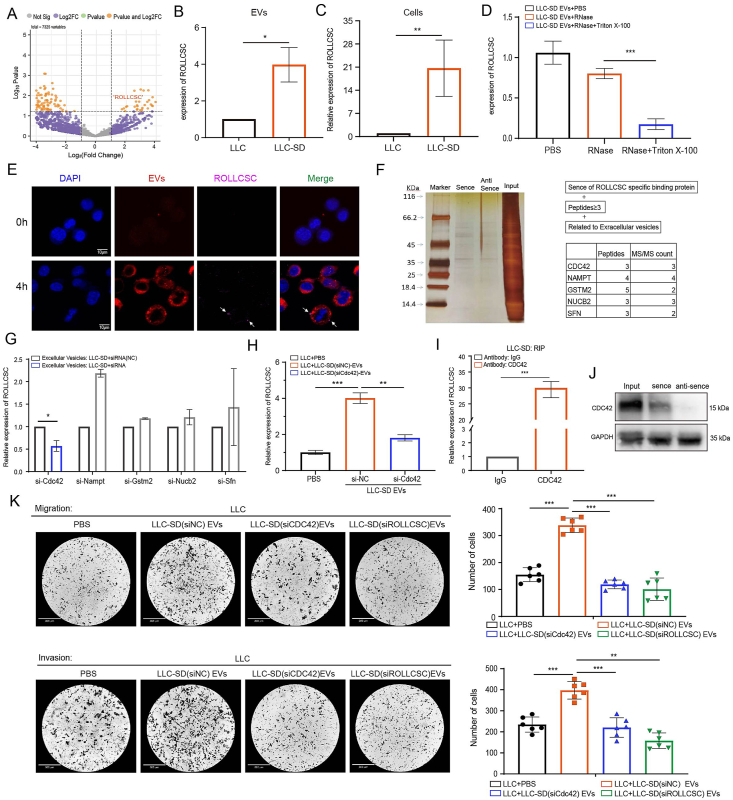
CDC42 facilitates the encapsulation of ROLLCSC into LLCSD extracellular vesicles. **(A)** Volcano plot of the differentially expressed genes (DEGs) in extracellular vesicles. Yellow indicates upregulation, while purple indicates downregulation. *P*-value <0.05, Log2|FC| > 1. **(B)** PCR analysis of the differential expression of ROLLCSC in extracellular vesicles (EVs). **(C)** PCR analysis of differential expression of ROLLCSC in cells. **(D)** PCR analysis of the effect of RNase and Triton X-100 treatment on the differential expression of ROLLCSC in EVs. **(E)** Confocal microscopy visualization of the colocalization of extracellular vesicles and ROLLCSC in cells. Red represents EVs, blue represents DAPI, and purple represents lncRNA. Scale bar = 10 μm. **(F)** Representative RNA pull-down silver staining and proteomic data for candidate proteins. **(G)** siRNA knockdown efficiency in LLC cells. **(H)** PCR analysis of the differential expression of ROLLCSC in extracellular vesicles. **(I)** RIP assay validating the interaction between ROLLCSC and CDC42, with IgG as a negative control. **(J)** Western blotting (WB) analysis confirming the differential interaction between the sense strand of ROLLCSC and CDC42. **(K)** Representative images and statistical analysis of transwell assay results for each group, with six randomly selected images per group for data analysis. (∗*p* < 0.05, ∗∗*p* < 0.01, ∗∗∗*p* < 0.001. ns, not significant. The results represent three independent experiments.)

We characterized the morphology (Fig. S1A) and stemness markers (Fig. S1B and 1C) of both cultured LLC and LLCSD cells as described in our previous study. In this study, we isolated and characterized EVs from LLC and LLCSD cells (Fig. S1D–F), confirming that the lncRNA ROLLCSC, which was expressed at significantly higher levels in LLCSD-derived EVs and cells (Fig. 1A–C), could be encapsulated into EVs and taken up by recipient LLC cells (Fig. 1D and E). We have constructed the overexpression and knockdown lentivirus of ROLLCSC (Fig. S1G and 1I). The specific encapsulation of lncRNAs into EVs may involve RNA-binding protein (RBP) transport.^28^ To further investigate this mechanism, we constructed both the sense and antisense chains of ROLLCSC. We screened EV-associated RBPs (including CDC42, NAMPT, GSTM2, NUCB1, and SFN; peptides>3) that specifically bound to the sense chain (Fig. 1F). We subsequently designed siRNAs to knock down these target RBPs (Fig. S1H). Our findings revealed that reduced expression of *Cdc42* significantly impacted ROLLCSC levels in LLCSD EVs (Fig. 1G), as did the amount of ROLLCSC that entered recipient LLC cells (Fig. 1H). RNA immunoprecipitation (RIP) and RNA pull-down experiments confirmed the specific binding between CDC42 and ROLLCSC (Fig. 1I and J). Finally, Transwell assays demonstrated that the decreased expression of both *Cdc42* and ROLLCSC in LLCSD exosomes significantly diminished the pro-metastatic effects mediated by EVs (Fig. 1K). These results indicated that CDC42 facilitates the encapsulation of ROLLCSC into LLCSD-derived EVs and subsequent entry into recipient LLC cells to enhance tumor metastasis.

### FTO-mediates m6A modification enhances ROLLCSC stability

**Figure 2:**
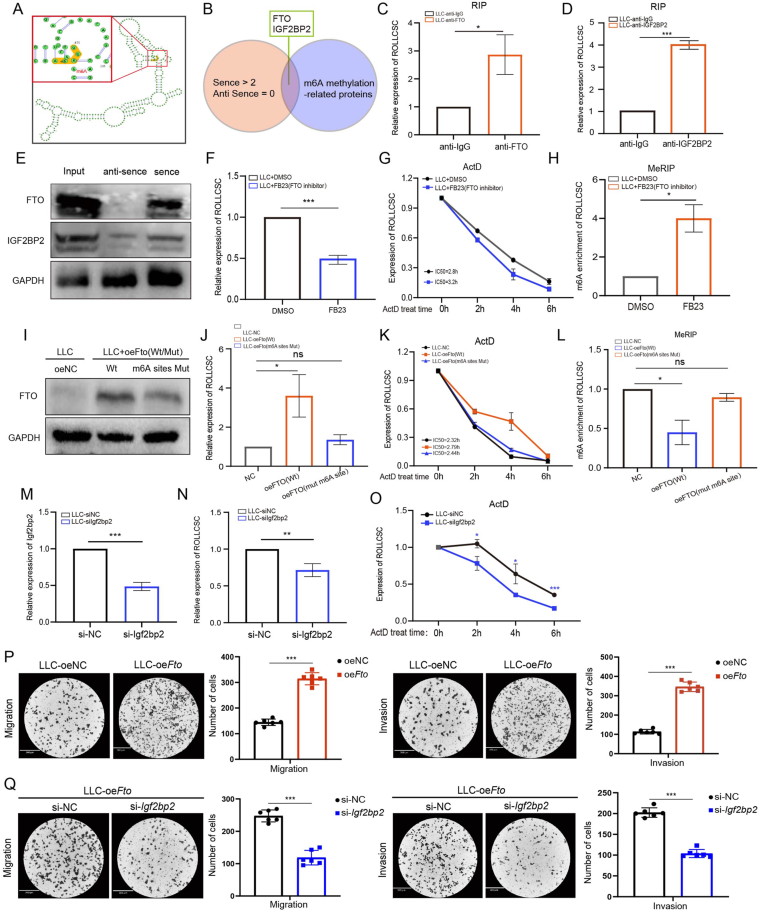
FTO-mediates m6A modification enhances ROLLCSC stability. **(A)** SRAMP analysis of the secondary structure of ROLLCSC and its potential m6A methylation sites. **(B)** Venn diagram showing methylation-related proteins specifically interacting with the sense strand. **(C** and **D)** RIP assay validating the interaction between ROLLCSC and the target protein, with IgG as a negative control. **(E)** Western blot confirming the specific interaction between ROLLCSC and the target protein. **(F)** Regulation of ROLLCSC expression by FTO inhibitor. **(G)** Effect of FTO inhibitor on the stability of the ROLLCSC gene, with ActD concentration at 2 μM. **(H)** MeRIP analysis of the impact of FTO inhibitor on the m6A methylation level of ROLLCSC. **(I)** Efficiency of lentiviral overexpression of FTO. **(J)** Regulation of ROLLCSC expression by lentiviral overexpression of FTO. **(K)** Effect of lentiviral FTO overexpression on the stability of the ROLLCSC gene, with ActD concentration at 2 μM. **(L)** MeRIP analysis of the impact of FTO inhibitor on the m6A methylation level of ROLLCSC. **(M)** Knockdown efficiency of si-*Igf2bp2*. (N) PCR analysis of the effect of si-*Igf2bp2* on ROLLCSC expression. (O) Effect of si-*Igf2bp2* on the stability of the ROLLCSC gene, with ActD concentration at 2 μM. (P–Q) Representative images and statistical analysis of transwell assay results for each group. Six randomly selected images per group were used for data analysis. (∗*p* < 0.05, ∗∗*p* < 0.01, ∗∗∗*p* < 0.001. ns, not significant. The results represent three independent experiments.)

Previous studies reported that m6A methylations could influence lncRNA stability.^29^, ^30^, ^31^ To investigate whether ROLLCSC could regulate m6A expression through methylations, we analyzed its secondary structure via the SRAMP online platform (http://www.cuilab.cn/m6asiteapp/result/sJ9QFa3pRT/) and identified potential m6A methylation sites (Fig. 2A). Furthermore, RNA pulldown LC‒MS/MS mass spectrometry analysis suggested that ROLLCSC may have specific binding interactions with methylation-related proteins (FTO and IGF2BP2) (Fig. 2B). FTO is recognized as a demethylase, whereas IGF2BP2 serves as a m6A reader protein that can recognize m6A modifications.^29^ These findings suggested that ROLLCSC might undergo m6A methylations mediated by the FTO/IGF2BP2 pathway. We subsequently observed that RIP (Fig. 2C and D) and RNA pulldown (Fig. 2E) assays confirmed a specific interaction between ROLLCSC and the methylation-related proteins FTO and IGF2BP2. As a classic demethylase, FTO is known to regulate lncRNA stability.^30^ We found that inhibiting *Fto* led to a marked reduction in ROLLCSC expression and stability while increasing its methylation levels (Fig. 2F–H). To further explore this phenomenon, we constructed both wild-type and mutant (m6A site mutations) *Fto*-overexpressing lentiviruses (Fig. 2I). The *Fto* (wild-type) lentivirus significantly increased ROLLCSC expression and stability while decreasing its methylation levels. In contrast, the *Fto* (mutant-type) lentivirus failed to produce these effects (Fig. 2J–L).

m6A reader proteins play essential roles in determining the fate of m6A-modified RNA by regulating its stability and degradation.^30^, ^31^, ^32^ Previous studies have shown that, in contrast to other m6A readers that promote RNA decay, such as YTHDCs, IGF2BPs tend to increase the stability of their target RNAs, thereby influencing gene expression.^32^ We constructed IGF2BP2-targeting siRNAs (Fig. 2M) and observed that when *Igf2bp2* expression was reduced, both the expression and stability of ROLLCSC significantly decreased (Fig. 2N–O). Finally, Transwell assays demonstrated that *Fto* overexpression significantly enhanced the migration and invasion capabilities of LLC cells, while si*Igf2bp2* was able to counteract this pro-metastatic effect (Fig. 2P and Q). Overall, these findings demonstrated that the stability of ROLLCSC was enhanced through demethylations mediated by FTO and IGF2BP2, thereby promoting its pro-metastatic efficiency in lung adenocarcinoma.

### The ROLLCSC/CDC42 positive feedback axis enhances the uptake of extracellular vesicles

**Figure 3:**
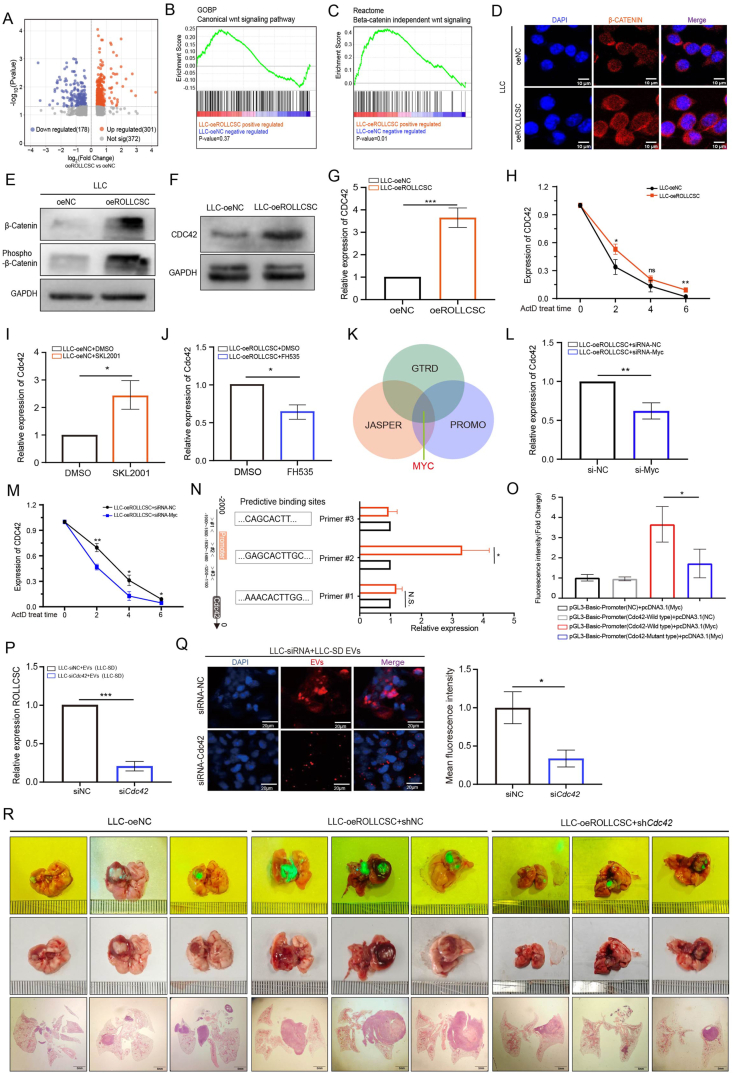
ROLLCSC promotes the expression of CDC42 via the Wnt/β-catenin signaling pathway. **(A)**Volcano plot showing the differentially expressed genes (DEGs) in LLC cells transfected with ROLLCSC overexpression lentivirus. Red indicates upregulation, blue indicates downregulation. *P*-value <0.05, Log2|FC| > 1. **(B–C)** GSEA analysis of the DEGs associated with ROLLCSC overexpression. **(D)** Confocal microscopy showing changes in β-catenin expression in the nucleus. **(E)** W B analysis validating the regulatory effect of ROLLCSC overexpression on β-catenin and phospho-β-catenin protein expression. **(F)** PCR analysis of the regulatory effect of ROLLCSC overexpression on *Cdc4*2 mRNA levels. **(G)** WB analysis of the regulatory effect of ROLLCSC overexpression on *Cdc42* protein levels. **(H)** Effect of ROLLCSC overexpression on *Cdc42* stability; ActD concentration = 2 μM. **(I–K)** PCR analysis of the regulatory effect of Wnt/β-catenin activator and inhibitor on *Cdc4*2 mRNA levels. **(L)** PCR analysis of the regulatory effect of Myc knockdown on *Cdc4*2 mRNA levels. **(M)** Effect of *Myc* knockdown on *Cdc4*2 mRNA stability. **(N)** ChIP assay validating the binding of three fragments within the *Cdc42* promoter region to anti-*Myc*. **(O)** Dual-luciferase assay validating the interaction between transcription factor *Myc* and the *Cdc42* promoter. The *Myc* plasmid was constructed using the pcDNA3.1 (+) vector, and both the wild-type and mutant *Cdc42* plasmids were constructed using the psicheck2 vector. **(P)** PCR analysis of ROLLCSC expression changes in LLC cells after co-culture with EVs. **(Q)** Representative images and statistical analysis of confocal microscopy showing the effect of CDC42 knockdown on LLC cell uptake of EVs. Blue represents DAPI, and red represents EVs. **(R)** Lung metastatic lesions were assessed by fluorescence imaging and H&E staining after euthanasia. Images are representative of three independent mice (*n* = 3). (∗*p* < 0.05, ∗∗*p* < 0.01, ∗∗∗*p* < 0.001. ns, not significant. The results represent three independent experiments.)

Our previous research demonstrated that ROLLCSC can activate the Wnt/β-catenin signaling pathway in melanoma. In this study, we further verified the regulatory relationship between ROLLCSC and the Wnt/β-catenin pathway in a lung adenocarcinoma model. We first performed high-throughput transcriptome sequencing. RNA-sequence analysis revealed 479 DEGs related to ROLLCSC in LLC cells (Fig. 3A). Through GSEA, we discovered significant enrichment of DEGs in the biological process (BP) and reactome pathways linked to the Wnt/β-catenin pathway (Fig. 3B and C).

In LLC cells, ROLLCSC overexpression promoted β-catenin nuclear translocation (Fig. 3D) and increased the protein expression of phospho-β-catenin and β-catenin (Fig. 3E). Additionally, the mRNA expression levels of the key mediators in the Wnt/β-catenin pathway were increased (Fig. S2A), indicating that ROLLCSC overexpression significantly activated the Wnt signaling pathway in LLC cells. Previous studies have reported that Wnt/β-catenin pathway activation can increase CDC42 expression; for example, the transcription factor Wnt5a can promote *Cdc42* transcription.^33^^,^^34^ We hypothesized that ROLLCSC influences *Cdc42* expression through the Wnt/β-catenin pathway. We found that ROLLCSC overexpression significantly increased the protein level, mRNA expression, and mRNA stability of *Cdc42* (Fig. 3F–H). Moreover, the expression of Wnt/β-catenin inhibitors and activators was also significantly correlated with *Cdc4*2 mRNA expression levels (Fig. 3I and J). We subsequently utilized the JASPAR, GTRD, and PROMO databases to predict Wnt/β-catenin pathway transcription factors that may bind to the *Cdc42* promoter region and conducted PCR validation (Fig. 3K; Fig. S2B and 2C). We discovered that *Myc* knockdown significantly reduced *Cdc42* expression and stability (Fig. 3M).

On the basis of the predicted binding sites in the *Cdc42* promoter region, we constructed three fragment primers, and ChIP experiments revealed that *Myc* specifically binds to the #2 promoter fragment of *Cdc42* (Fig. 3N). Dual-luciferase reporter assays further confirmed the direct binding of the transcription factor *Myc* to the *Cdc42* promoter region (Fig. 3O). The literature reports that *Cdc42* can further promote the uptake of EVs by recipient cells.^35^ We found that *Cdc42* knockdown reduced LLC cell uptake of LLCSD-derived EVs (Fig. 3P and Q), and the pro-metastatic effect mediated by LLCSD-derived EVs was reversed by sh*Cdc42* lentivirus both *in vitro* and *in vivo* (Fig. 3R and Fig. S2D). These results demonstrated that ROLLCSC activates the Wnt/β-catenin signaling pathway to facilitate *Cdc42* transcription via the transcription factor MYC. The upregulation of *Cdc42* further enhances the uptake of EVs by recipient LLC cells, creating a positive feedback loop between the LLCSD and non CSC LLCs in the TME that promotes lung adenocarcinoma metastasis.

### ROLLCSC-mediated lipid metabolism remodeling resists ferroptosis

**Figure 4:**
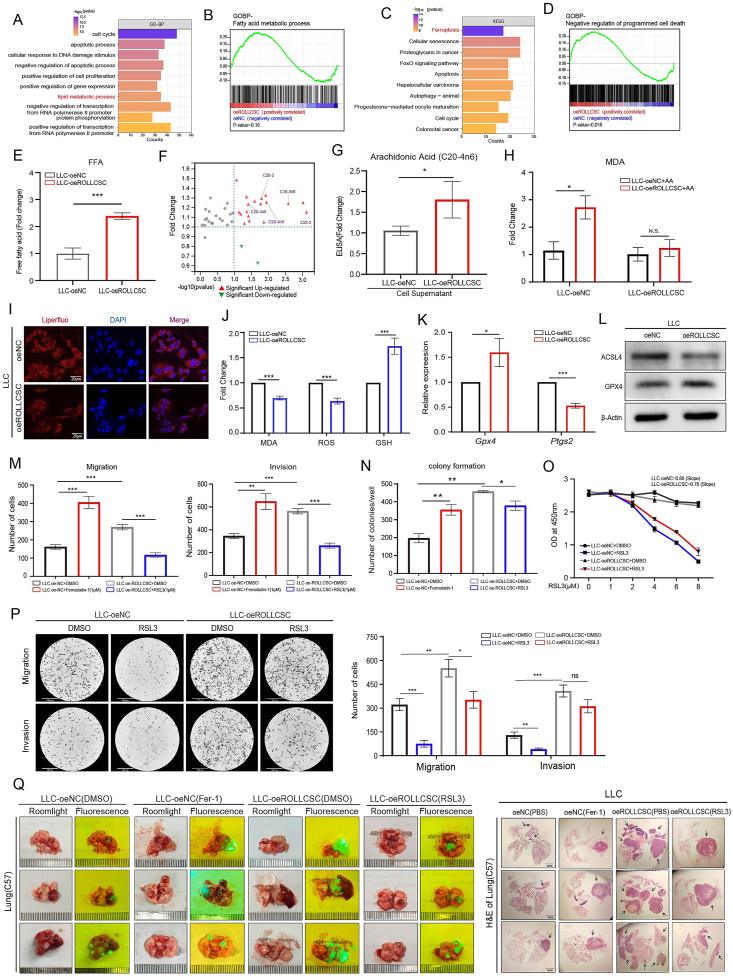
ROLLCSC promotes tumor metastasis by inhibiting ferroptosis. **(A)** Gene Ontology Biological Process (GO/BP) enrichment analysis of differentially expressed genes (DEGs) in RNA-seq data was performed using DAVID to obtain functional insights. **(B)** Gene Set Enrichment Analysis (GSEA, version 4.2.3) was conducted on DEGs from RNA-seq data to evaluate gene set enrichments. **(C)** KEGG pathway enrichment analysis of RNA-seq DEGs was performed using DAVID to identify significantly enriched pathways. **(D)** GSEA (version 4.2.3) was performed on RNA-seq DEGs to assess gene set enrichments. **(E)** The free fatty acid content in LLC cells was quantitatively measured using a specialized free fatty acid detection kit, as detailed in the methods section. **(F)** Targeted free fatty acid analysis by GC/MS was conducted in LLC cells, with the differential expression of free fatty acids visualized in the ROLLCSC overexpression group. Three biological replicates were performed each group. **(G)** The concentration of arachidonic acid in the supernatant of LLC cells was quantified using ELISA. **(H)** The MDA levels in each group LLC cells following the addition of arachidonic acid. **(I)** Representative confocal microscopy images showing changes in Liperfluo levels in each group. Red represents Liperfluo, blue represents DAPI. **(J)** The regulatory effect of ROLLCSC overexpression on MDA, ROS, and GSH levels in LLC cells. **(K)** mRNA expression levels of ferroptosis markers in LLC cells. **(L)** Protein expression levels of ferroptosis markers in LLC cells. **(M)** Statistical analysis of transwell assay results for each group; Left panel: Migration; Right panel: Invasion; Six randomly selected images from each group were used for data analysis. **(N)** Statistical analysis of colony formation assay results for each group. **(O)** Absorbance at 450 nm after 2 h of CCK-8 treatment in LLC cells in each group following RSL3 addition. **(P)** Representative images and statistical analysis of transwell assay results for each group. Six randomly selected images per group were used for data analysis. **(Q)** Lung metastatic lesions were assessed by fluorescence imaging and H&E staining after euthanasia. Images are representative of three independent mice (*n* = 3). (∗*p* < 0.05, ∗∗*p* < 0.01, ∗∗∗*p* < 0.001. ns, not significant. The results represent three independent experiments.)

To gain a comprehensive understanding of the downstream regulation of ROLLCSC, a series of enrichment analyses were conducted on ROLLCSC-related DEGs (Fig. 3A). These DEGs could be enriched in lipid and fatty acid metabolic processes according to the GOBP and GSEA analyses (Fig. 4A and B), which is in line with our previous findings. Furthermore, Kyoto Encyclopedia of Genes and Genomes (KEGG) and gene set enrichment analyses (GSEAs) suggested that ROLLCSC-related DEGs were related to ferroptosis (Fig. 4C and D). The interaction between ferroptosis and lipid metabolism is primarily mediated by free fatty acids (FFAs), particularly the PUFAs, which are believed to be the primary drivers of dysregulated lipid-induced ferroptosis. We observed that ROLLCSC could increase the accumulation of FFAs (Fig. 4E), and FFA-targeted GC/MS further confirmed that ROLLCSC stimulated an increase in FFA subclasses (SFAs, MUFAs, and PUFAs) (Fig. S3A). Notably, the levels of polyunsaturated fatty acids (FAs), which drive lipid production via ferroptosis, were also significantly elevated (Fig. 4F). To verify the GC/MS results, we selected arachidonic acid (AA), the most representative constituent of PUFA, for ELISA experiments, and the results showed that ROLLCSC overexpression indeed drove an increase in AA (Fig. 4G). The peroxidation-prone nature of PUFA-PLs serves as a crucial factor in inducing ferroptosis, which also affects the metastatic capacity of tumor cells. We hypothesized that an increased concentration of PUFAs affects the ferroptosis and metastatic potential of LUAD cells. The evaluation of ferroptosis indicators revealed a significant increase in lipid peroxidation (malondialdehyde, MAD) levels in LLC cells after AA treatment; however, this trend was not significant in ROLLCSC-overexpressing cells (Fig. 4H). Additionally, transwell assays revealed that AA-treated cells in the control group presented a reduced tumor metastatic capacity, whereas this trend was not observed in the ROLLCSC-overexpressing group (Fig. S3B). A cell viability assay revealed that ROLLCSC exhibited the highest sensitivity to a ferroptosis inhibitor (Fer-1), as opposed to an autophagy inhibitor (BafA1) or an apoptosis inhibitor (Z-VAD-FMK) (Fig. S3C). The most direct phenotype of ferroptosis is enhanced cellular lipid peroxidation, and an appreciable decrease in the level of peroxidation (red; EX = 488 nm, EM = 525 nm) was observed in LLC cells upon upregulation of ROLLCSC (Fig. 4I). Moreover, the upregulation of ROLLCSC resulted in a marked reduction in MDA and ROS levels, accompanied by a substantial increase in the abundance of the crucial antioxidant glutathione (GSH) (Fig. 4J). We subsequently confirmed the significant regulatory effect of ROLLCSCs on ferroptosis markers through qPCR (Fig. 4K) and WB analysis (Fig. 4L). Through *in vitro* experiments, including migration, invasion (Fig. 4M; Fig. S3D), and colony formation (Fig. 4N; Fig. S3E), we showed that both the addition of a ferroptosis inhibitor (Fer-1) and ROLLCSC upregulated induced a pro-metastatic phenotype in LLC cells; furthermore, the introduction of a ferroptosis inducer (RSL3) in LLC-oeROLLCSC cells reverses this pro-metastasis effect. Cell viability assays indicated that the upregulated ROLLCSC attenuated the susceptibility of LLC cells to ferroptosis (Fig. 4O). In addition, the transwell assays (Fig. 4P) and colony formation assays (Fig. S3F, G) revealed that the addition of RSL3 effectively suppressed the metastatic colonization abilities of LLC cells. As expected, the upregulated ROLLCSC group exhibited greater resistance to the ferroptosis inducer compared to the control group. This resistance of ferroptosis appeared to confer protection to the cells, alleviating the declining trend of their ability to metastatic colonization (Fig. S3H and 3I). To verify our findings *in vivo*, we established an orthotopic lung metastasis model in C57BL/6 mice. We noted that administering an intraperitoneal injection of ferrostatin-1 (Fer-1) to control group mice significantly increased lung metastasis *in vivo*, similar to the effects observed in the ROLLCSC-overexpressing group. Conversely, when the ROLLCSC-overexpressing group of mice received an intraperitoneal injection of RSL3, the extent of lung metastasis was mitigated (Fig. 4Q). Our results indicated that upregulating ROLLCSC reduces ferroptosis sensitivity during LUAD metastasis both *in vitro* and *in vivo*.

### ROLLCSC attenuates ferroptosis susceptibility in lung adenocarcinoma by regulating the ELOC/ACSL4 axis

**Figure 5:**
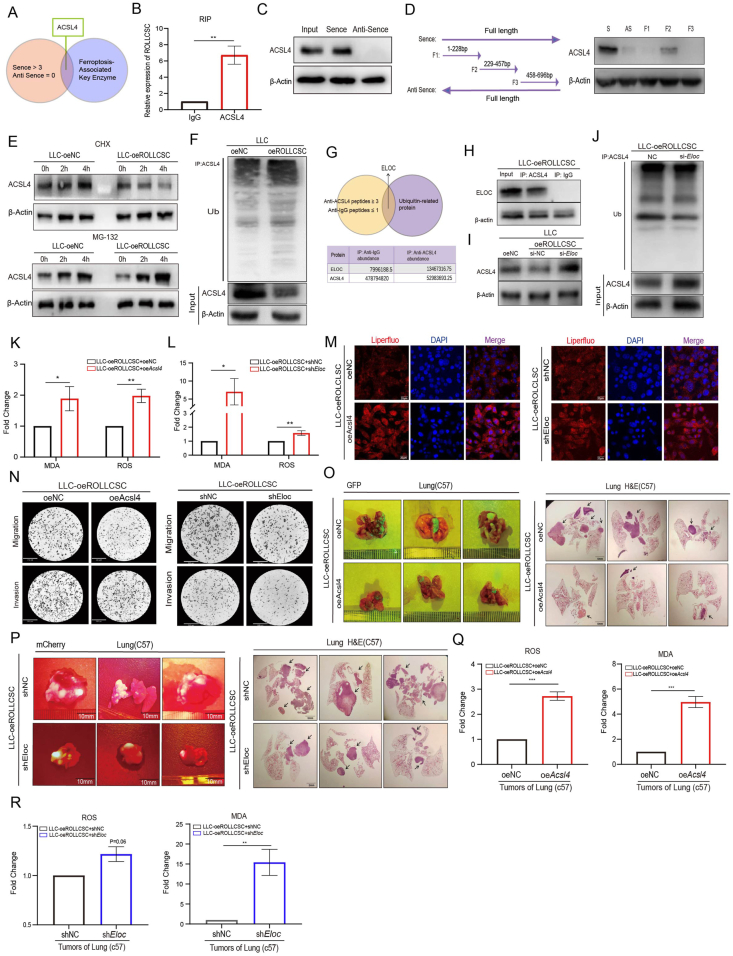
ACSL4/ELOC mediates the ferroptosis-inhibiting process of ROLLCSC in tumor cells. **(A)** Venn diagram showing methylation-related proteins specifically interacting with the sense strand. **(B)** RIP verified the direct binding of anti-ACSL4 and ROLLCSC, and anti-IgG served as a negative control. **(C)** WB experiments identified the direct binding of anti-ACSL4 and ROLLCSC. **(D)** WB experiments identified the direct binding of anti-ACSL4 and ROLLCSC fragments. **(E)** Western Blot was employed to examine the influence of ROLLCSC on the protein level of ACSL4 subsequent to MG132 (1 μM) and CHX (1 μM) treatment. **(F)** Western blot of the total ubiquitination proteins and ACSL4 from control and ROLLCSC overexpression of lentivirus transfection LLC cells. **(G)** Immunoprecipitation and mass spectrometry were used to screen the anti-ACSL4 interacting proteins in LLC-oeROLLCSC cells. **(H)** Western blot confirmed the direct binding of ACSL4 to ELOC in LLC-oeROLLCSC cells. **(I)** Western blot was used to detect the protein expression of ACSL4 after transfection of si-Eloc in LLC-oeROLLCSC cells. **(J)** Western blot was used to detect the expression of ACSL4 protein and total ubiquitin protein after transfection of si-Eloc in LLC-oeROLLCSC cells. **(K)** Investigated the effect of Acsl4 overexpression on MDA and ROS levels in LLC-oeROLLCSC cells. **(L)** Investigated the effect of Eloc knockdown on MDA and ROS levels in LLC-oeROLLCSC cells. **(M)** Liperfluo reagent was used to verify the effect of Acsl4 overexpression and Eloc knockdown on intracellular lipid peroxidation levels in LLC-oeROLLCSC cells; Red = intracellular lipid peroxidation, DAPI = nucleus. **(N)** Representative images of transwell results suggest the effect of *Acsl4* and *Eloc* on the migration (upper) and invasion (down) ability of LLC cells. **(O**, **P)** Lung metastatic lesions were assessed by fluorescence imaging and H&E staining after euthanasia. Images are representative of three independent mice (*n* = 3). **(Q)** Investigated the effect of Acsl4 overexpression on ROS and MDA levels in lung metastases of C57BL/6 mice. **(R)** Investigated the effect of *Eloc* knockdown on ROS and MDA levels in lung metastases of C57BL/6 mice. (∗*p* < 0.05, ∗∗*p* < 0.01, ∗∗∗*p* < 0.001. ns, not significant. The results represent three independent experiments.)

Cytoplasmic lncRNAs can regulate cancer progression by interacting with RNA-binding proteins (RBPs).^28^ To understand the mechanistic function of ROLLCSC in ferroptosis, we utilized the pull-down technique to screen for proteins that interact with ROLLCSC (Fig. 5A; Fig. S4A). ACSL4, a critical protein involved in ferroptosis, is widely recognized as a promoter of ferroptosis, as it facilitates the incorporation of PUFAs into phospholipids, leading to lipid peroxidation-based ferroptosis.^36^ RIP (Fig. 5B) and RNA pulldown (Fig. 5C) assays confirmed the binding relationship between ROLLCSC and ACSL4. We subsequently constructed and biotinylated three fragments of ROLLCSC (F1: 1–228 bp, F2: 229–457 bp, and F3: 458–696 bp). These fragments were then used in the pull-down assay with LLC cell lysates. We found that the 2nd fragment of ROLLCSC mediated the interaction with ACSL4 (Fig. 5D). LncRNAs regulate protein degradation or generation by influencing the posttranslational modifications of RBPs, including ubiquitination, phosphorylation, acetylation, and other processes. ACSL4 is reported to have ubiquitination modification sites. We thus hypothesized that the underlying mechanism by which ROLLCSC negatively regulates ACSL4 protein is related to ubiquitination (Fig. 4L). Subsequently, we added CHX and MG132 to both the control group and ROLLCSC-overexpressing group to examine ACSL4 protein expression (Fig. 5E). WB analysis suggested that ROLLCSC may regulate ACSL4 expression through the ubiquitin–proteasome system (Fig. 5F).

To investigate the mechanism by which ROLLCSC enhances the ubiquitin‒proteasome degradation of ACSL4, we conducted immunoprecipitation and mass spectrometry analyses on LLC cells overexpressing ROLLCSC and controls to precisely identify the proteins that specifically interact with ACSL4 (anti-ACSL4 peptide ≥3, anti-IgG peptide ≤1). By integrating the peptides and protein expression data, we discovered an E3 ubiquitin ligase, ELOC, with a higher binding affinity for ACSL4 in ROLLCSC overexpression cells (Fig. 5G and H). The knockdown of *Eloc* in ROLLCSC-overexpressing cells rescued both the protein expression (Fig. 5I) and ubiquitination levels of ACSL4 (Fig. 5J). In summary, our findings suggest that the lncRNA ROLLCSC may serve as a scaffold, facilitating the interaction between the E3 ubiquitin ligases ELOC and ACSL4, thereby accelerating the degradation of ACSL4.

To explore the role of the ELOC/ACSL4 complex in resisting ferroptosis, we transfected Acsl4-overexpressing (oe*Acsl4*) and *Eloc-knockdown* (sh*Eloc*) lentiviruses to validate this hypothesis (Fig. S4B and 4C). Notably, oe*Acsl4* and sh*Eloc* significantly reversed the ferroptosis levels attenuated by ROLLCSC, as confirmed by the MDA, ROS (Fig. 5K and L), and Liperfluo assays (Fig. 5M). *In vitro* experiments revealed that oe*Acsl4* and sh*ELoc* significantly reversed the ROLLCSC-mediated prometastatic effect, as evidenced by Transwell (Fig. 5N; Fig. S4D and 4E) and colony formation (Fig. S4H and 4I) assays. *In vivo* experiments demonstrated that oe*Acsl4* (Green) and sh*Eloc* (Red) resulted in a significant reduction in metastatic tumor foci in the lungs of the mice (Fig. 5O and P; Fig. S4J and 4K), accompanied by an increase in ferroptosis indicators (ROS, MDA) within these foci (Fig. 5Q and R; Fig. S4L, 4J and 4K).

In summary, these results indicate that ROLLCSC, which acts as a scaffold, facilitates the interaction between the E3 ubiquitin ligases ELOC and ACSL4. This interaction significantly affects the sensitivity of LLC cells to ferroptosis, ultimately accelerating metastasis in lung adenocarcinoma.

### ROLLCSC acts as a ceRNA to improve intra-mitochondrial GSH transport by regulating *Slc25a11*

**Figure 6:**
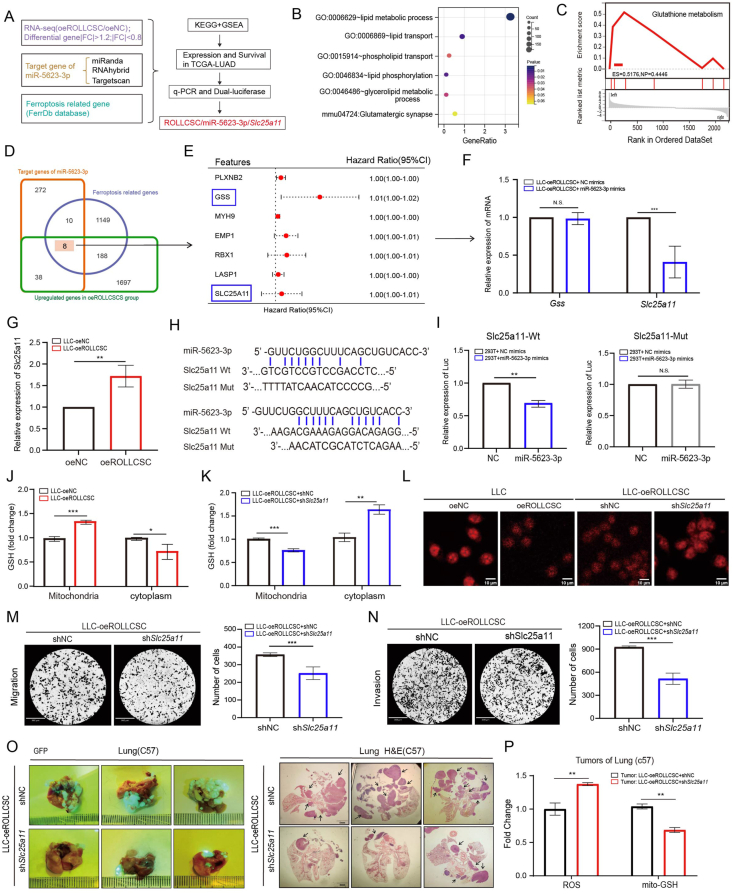
ROLLCSC acts as a ceRNA to promote SLC25A11. **(A)** Schematic diagram of the construction of ceRNA of ROLLCSC network. **(B)** The target genes of miR-5623-3p were enriched by KEGG and GO analysis; Only partial data of lipid-related pathways are shown. **(C)** The target genes of miR-5623-3p were combined with TCGA-LUAD data to perform GSEA analysis (Sangerbox; http://vip.sangerbox.com/home.html). **(D)** Multiple datasets further predict target genes of miR-5623-3p, visualized by Venn diagram. **(E)** Survival analysis of target gene (D) in TCGA-LUAD data and visualized by Sangerbox. **(F)** PCR detected the regulatory effect of miR-5623-3p on *Gss* and *Slc25a11*.**(G)** PCR detected the regulatory effect of ROLLCSC on Slc25a11. **(H)** The wild-type and mutant-type plasmids of Slc25a11 in Dual-luciferase. **(I)** Dual luciferase assay detected the direct binding of *Slc25a11* to miR-5623-3p. **(J)** Effect of ROLLCSC on GSH in mitochondria and cytoplasm of LLC cells. **(K)** Effect of *Slc25a11* on GSH in mitochondria and cytoplasm of LLC-oeROLLCSC cells. **(L)** Effect of ROLLCSC and *Slc25a11* on mito-ROS (red fluorescence) in LLC cells. **(M)** Representative images and statistical analysis of transwell results suggest the effect of *Acsl4* overexpression on the migration (upper) of LLC cells. Six randomly selected images per group were used for data analysis. **(N)** Representative images and statistical analysis of transwell results suggest the effect of *Acsl4* overexpression on the migration (upper) of LLC cells. Six randomly selected images per group were used for data analysis. **(O)** Lung metastatic lesions were assessed by fluorescence imaging and H&E staining after euthanasia. Images are representative of three independent mice (*n* = 3). **(P)** Effect of *Slc25a11* on ROS and mito-GSH in lung metastases from C57BL/6 mice, number of samples (*n* = 3). (∗*p* < 0.05, ∗∗*p* < 0.01, ∗∗∗*p* < 0.001. ns, not significant. The results represent three independent experiments.)

The regulatory mechanism of lncRNAs in tumor progression involves not only binding to RBPs but also another well-established mechanism: acting as ceRNAs (competing endogenous RNAs), which target miRNAs and subsequently affect downstream target genes.^9^ Our previous research revealed that ROLLCSC can directly bind to miR-5623-3p; however, the potential mechanism by which ROLLCSC affects the downstream targets of miR-5623-3p remains unclear.^11^ We determined that ROLLCSC can directly bind to and negatively regulate miR-5623-3p (Fig. S5A and 4B). We then combined bioinformatics analysis with experimental validation to explore the downstream regulatory mechanisms, as outlined in the flowchart shown in Figure 6A. The downstream target genes of miR-5623-3p were predicted through the integration of multiple databases (TargetScan, miRanda, and RNAhybrid) (Fig. S5C). KEGG and GO analyses revealed that these target genes were associated with lipid metabolic, lipid transport and glutamatergic synapse-related biological processes (Fig. 6B). In addition, these genes were introduced into the TCGA-LUAD database for differential gene set enrichment analysis (GSEA), and the results revealed that these target genes were positively correlated with the glutathione metabolism pathway (Fig. 6C). Next, we conducted an cross analysis of the three datasets encompassing RNA-seq data, miRNA target genes, and ferroptosis-related genes (Fig. 6D). By integrating these datasets with the TCGA-LUAD dataset, we successfully identified several ferroptosis-related genes associated with a poor prognosis (PLXNB2, MYH9, EMAP1, GSS, MYH9, EMAP1, RBX1, LASP1, and SLC25A11) (Fig. 6E). It has been reported that GSS and SLC25A11 play critical roles in glutathione metabolism. *Gss* facilitates the production of GSH, whereas *Slc25a11* promotes the transportation of GSH within mitochondria.^14^ Q-PCR experiments revealed that *Slc25a11* is negatively regulated by miR-5623-3p in LLC cells; however, such regulation was not observed in Gss (Fig. 6F). Furthermore, overexpression of ROLLCSC resulted in significant upregulation of *Slc25a1*1 mRNA expression (Fig. 6G). Dual luciferase assays confirmed that *Slc25a11* could directly bind to miR-5623-3p (Fig. 6H and I). These results indicated that ROLLCSC could act as a ceRNA to promote *Slc25a1*1 mRNA expression by targeting miR-5623-3p. *Slc25a11* has been reported to inhibit ferroptosis in tumor cells by facilitating the transport of GSH into mitochondria. To further investigate whether ROLLCSC alters mitochondrial GSH transport through *Slc25a11*, we isolated mitochondria and cytoplasm and observed that the overexpression of ROLLCSC led to an increase in mitochondrial GSH levels, accompanied by a decrease in cytoplasmic GSH levels (Fig. 6J); this phenomenon could be rescued by knocking down *Slc25a11* (Fig. 6K; Fig. S5D). The intracellular mitochondrial ROS level was assayed via a Mito-ROS probe. Confocal microscopy revealed that the overexpression of ROLLCSC notably attenuated mitochondrial ROS levels. Conversely, the knockdown of *Slc25a11* significantly reversed this suppressive effect (Fig. 6L; Fig. S5E). Additionally, we found that knocking down *Slc25a11* reversed the ROLLCSC-mediated promotion of cell migration, invasion (Fig. 6M−N), and colony formation (Fig. S5F), as well as tumor metastasis *in vivo* (Fig. 6O; Fig. S5G). Furthermore, these results were consistent with the results observed *in vitro*. The downregulated *Slc25a11*chould affected ROS and GSH levels in lung metastases of C56BL/6 mice (Fig. 6P; Fig. S5H and 5I).

### ROLLCSC and its downstream targets are of prognostic significance in patients of lung adenocarcinoma

**Figure 7:**
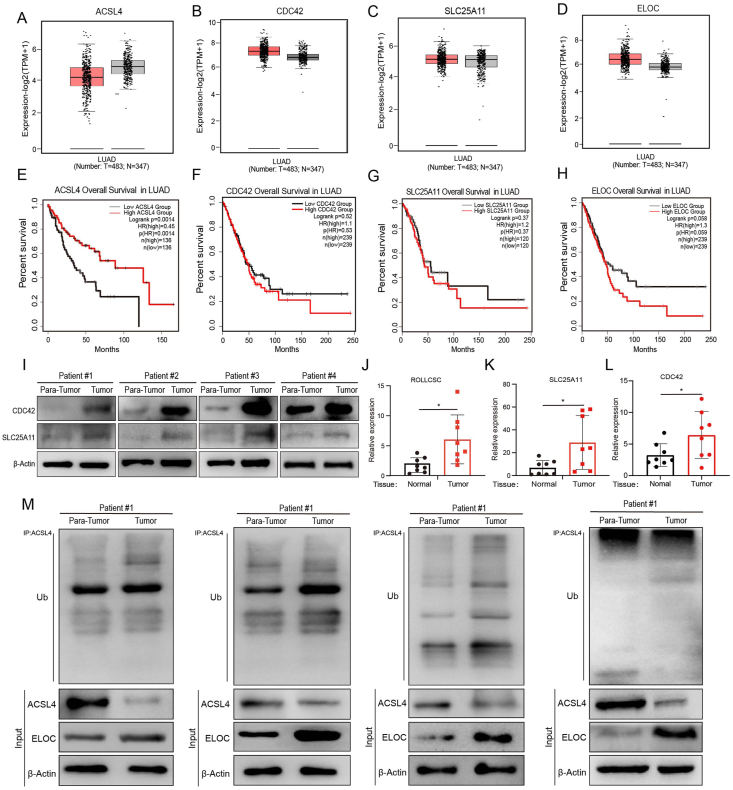
ROLLCSC attenuates ferroptosis by inhibiting mitochondrial lipid peroxidation via activation of *Slc25a11*. **(A–D)** The differential expression of Target genes in tumor patients, statistics from the TCGA-LUAD database, and visualized using GEPIA 2.0. **(E–H)** The survival analysis of Target genes in lung adenocarcinoma patients, visualized through GEPIA 2.0. **(I)** Western Blot detected the differential expression of SLC25A11 and CDC42 in tumor tissues and adjacent tissues of patients with lung adenocarcinoma; number of samples (*n* = 4). **(J)** PCR detected the differential expression of ROLLCSC in tumor tissues and adjacent tissues of patients with lung adenocarcinoma; number of samples (*n* = 8). **(K)** PCR detected the differential expression of SLC25A11 in tumor tissues and adjacent tissues of patients with lung adenocarcinoma; number of samples (*n* = 8). **(L)** PCR detected the differential expression of CDC42 in tumor tissues and adjacent tissues of patients with lung adenocarcinoma; number of samples (*n* = 8). **(M)** Western Blot detected the ubiquitination modification of ACSL4 immunoprecipitation assay and the differential expression of ACSL4 and ELOC at the protein level; number of samples (*n* = 4). (∗*p* < 0.05, ∗∗*p* < 0.01, ∗∗∗*p* < 0.001. ns, not significant. The results represent three independent experiments.)

To ascertain whether the DEGs identified in our study have the potential to serve as prognostic markers in LUAD, we initially obtained the standardized TCGA-LUAD dataset from the UCSC database (https://xenabrowser.net/), which includes 483 tumor samples and 347 normal samples. Subsequently, we extracted gene expression data for ENSG00000070831 (CDC42), ENSG00000068366 (ACSL4), ENSG00000154582 (ELOC), and ENSG00000108528 (SLC25A11) from each sample. Notably, according to GEPIA 2.0, ACSL4 expression (Fig. 7A) was lower than that of CDC42 (Fig. 7B), SLC25A11 (Fig. 7C), and ELOC (Fig. 7D) and was greater in patients with lung adenocarcinomas. Furthermore, we investigated the impact of these genes on the overall survival of LUAD patients. Our findings revealed that high expression of ACSL4 is associated with a favorable prognosis (Fig. 7E), whereas increased expression levels of CDC42 (Fig. 7F), SLC25A11 (Fig. 7G), and ELOC (Fig. 7H) are indicative of significantly poorer outcomes.

More importantly, we obtained tumor and para-tumor tissues from patients diagnosed with LUAD via clinicopathological biopsy and extracted RNA and protein from these tissues. Q-PCR analysis revealed that the expression of ROLLCSC, CDC42, and SLC25A11 was increased in lung adenocarcinoma patients compared to non-tumor patients (Fig. 7I). Similarly, the protein levels of CDC42, MYC, SLC25A11, exhibited a consistent trend (Fig. 7J–L). Subsequently, through immunoprecipitation experiments conducted on tumor tissues and para-tumor tissues of LUADs, we observed that the ubiquitination of ACSL4 was enhanced and its protein expression was reduced in the tumor tissues. Additionally, the expression of ELOC protein was correspondingly elevated in the tumor tissues (Fig. 7M). Clinically, these results confirmed that the orchestrated cascade reaction encompassing ROLLCSC and targets that we have characterized in the experimental LUAD models, plays a crucial role in the development of effective antitumor therapies for lung adenocarcinoma.

## Discussion

Extracellular vesicles (EVs) act as “messengers” of intercellular communication by delivering their biologically active components (lipids, proteins, or nucleic acids) to nearby or distant cells.^8^ Tumor cell-secreted EVs can modify the extracellular matrix (ECM) or stromal cells to promote metastasis.^6^ Previous studies have shown that tumor-derived EVs can influence tumor progression in the tumor microenvironment (TME) by affecting nearby tumor cells.^37^^,^^38^ Our previous research identified a specific phenotype in which high-metastatic LUAD stem cells (LLCSDs) transfer their “metastatic capability” to low-metastatic LUAD non-stemness cells (LLCs) through EVs, promoting tumor progression.^37^ However, little is known about the mechanisms by which EVs mediate the transfer of “metastatic capability” between tumor cells.

Cell division cycle 42 (CDC42) encodes a small GTPase of the Rho subfamily that regulates filopodium formation and mediates cytoskeletal reorganization to promote cell migration, morphology, and endocytosis.^39^ Previous studies have demonstrated that CDC42 is highly expressed in EVs and can enter recipient cells along with EVs to maintain their function.^35^^,^^40^ For example, CDC42 could be transported by CRC-EVs to macrophages to activate NOD1,^41^ meanwhile M1 macrophages secrete CDC42 to adjacent macrophages, further enhancing their phagocytosis through positive feedback.^40^ In our study, we found that CDC42 is not only transported by extracellular vesicles (EVs) but also acts as an RNA-binding protein (RBP), promoting the binding of RNA to EVs and their entry into recipient cells. In LLCSD cells, CDC42 directly binds to ROLLCSC, facilitating its encapsulation into EVs and subsequent uptake by recipient LLC cells. ROLLCSC enhances the transcriptional activation of CDC42 by the transcription factor Myc through Wnt/β-catenin signaling pathway activation, thereby augmenting the uptake of EVs by recipient LLC cells via a positive feedback loop (Fig. 2).

Compared to epigenetic modifications that regulate gene expression at the transcriptional level, such as DNA methylation and histone acetylation, m6A modification has significantly advanced our understanding of post-transcriptional gene regulation in eukaryotic cells.^32^^,^^42^ Tumor cells, under various conditions, maintain self-renewal, proliferation, and metastasis by coordinating abnormal m6A modifications of specific RNAs.^43^ FTO, the first identified m6A demethylase, plays a key role in regulating processes such as adipocyte differentiation and cancer cell proliferation and metastasis by modulating m6A modification levels.^44^^,^^45^ FTO mediates the demethylation of m6A on mRNA or lncRNA and, in cooperation with m6A reader proteins, regulates the stability of m6A-modified RNA. For example, FTO-mediated demethylation of APOE mRNA is recognized and stabilized by the m6A reader IGF2BP2.^46^ FTO reduces m6A methylation on LINC00022, inhibiting its degradation through the m6A reader YTHDF2.^30^ The cellular functions of m6A-modified RNAs are primarily determined by m6A reader proteins. IGF2BP2, unlike the YTH domain family proteins that mediate RNA degradation, stabilizes m6A-modified RNA.^47^ For instance, Li et al demonstrated that WTAP-mediated m6A modification of DIAPH1-AS1 enhances its stability via an IGF2BP2-dependent pathway.^48^ Both FTO and IGF2BP2 are key members of the m6A regulatory machinery and are involved in stabilizing lncRNAs. However, studies on the FTO/IGF2BP2 axis in regulating lncRNA stability remain limited. In this study, RNA pulldown and proteomic analyses revealed that ROLLCSC directly interacts with FTO and IGF2BP2. FTO mediates the m6A demethylation of the lncRNA ROLLCSC, which is recognized by the m6A reader IGF2BP2, thereby enhancing the stability of ROLLCSC.

Abnormal alterations in cellular metabolism, particularly lipid metabolism, are well-established hallmarks of cancer. Cancer cells frequently exhibit enhanced lipid metabolism to secure a critical energy source for their abnormal proliferation and metastasis.^12^^,^^17^ For example, Lu et al demonstrated that PCK1 augments lipid synthesis capacity via the INSIG1/2-SREBP signaling pathway, thereby promoting hepatocellular carcinoma development.^49^ Furthermore, inhibition of fatty acid supply has been shown to impede tumor progression, with fatty acid synthase inhibitors effectively inhibiting tumor progression.^50^^,^^51^ In our previous research, we discovered that the lncRNA ROLLCSC induces lipid metabolic reprogramming, resulting in elevated intracellular free fatty acids in LUAD cells, which in turn enhances tumor metastasis.^11^ Our findings indicate that free fatty acids, especially polyunsaturated fatty acids, have a role in promoting ferroptosis. PUFA-PLs are extremely susceptible to lipid peroxidation because of their numerous double bonds, making them crucial metabolic substrates in ferroptosis.^20^ However, the question remains regarding how lung adenocarcinoma cells effectively counteract the potential risk of ferroptosis during abnormal fatty acid metabolism. In this study, we identified a functional lncRNA, ROLLCSC, which functions as a homeostatic regulator of ferroptosis and abnormal lipids. ROLLCSC effectively inhibits ferroptosis susceptibility and promotes LUAD progression by inhibiting ACSL4 and activating SLC25A11.

## Conclusion

In this study, we revealed the mechanism by which EVs lncRNAs facilitate the transfer of “metastatic capability” between LUAD stem cells (LLC-SD) and LUAD non-stem cells (LLC). Specifically, CDC42 promotes the encapsulation of ROLLCSC into LLCSD EVs and its subsequent entry into recipient LLC cells. ROLLCSC enhances *Cdc42* transcriptional activation mediated by the transcription factor *Myc* in LLC cells, which in turn enhances the uptake of EVs by recipient LLC cells through a positive feedback loop. Within recipient LLC cells, the FTO-mediated m6A modification of ROLLCSC is recognized by the m6A reader IGF2BP2, thereby increasing its stability. This stabilization allows ROLLCSC to more effectively reduce ferroptosis susceptibility in LUADs by inhibiting ACSL4 and activating *Slc25a11*, consequently promoting tumor progression.

## CRediT authorship contribution statement

**Yu-Han Zhang:** Writing – original draft, Visualization, Validation, Software, Project administration, Methodology, Investigation, Formal analysis, Conceptualization, Methodology, Conceptualization. **Jia-Cheng Xie:** Methodology, Formal analysis, Data curation. **Ting Ye:** Resources. **Shi-Meng Guo:** Resources, Data curation. **Xue Han:** Methodology, Investigation, Conceptualization. **Si Yang:** Methodology, Investigation. **Lei Shi:** Methodology, Data curation. **Yi-Shi Li:** Resources, Methodology. **H. Rosie Xing:** Writing – review & editing, Funding acquisition, Data curation, Conceptualization. **Jing-Yu Li:** Writing – review & editing, Funding acquisition, Data curation. **Jian-Yu Wang:** Writing – review & editing, Investigation, Funding acquisition, Data curation, Conceptualization.

## Ethics approval and consent to participate

Animal experiments and LUAD patient samples were conducted in strict adherence to the biomedical ethics review guidelines of Chongqing Medical University, all experiments were performed following relevant guidelines and regulations.

## Consent for publication

All the authors agreed to the manuscript’s publication.

## Availability of data and materials

The data that support the findings of this study are available from the corresponding authors upon reasonable.

## Declaration of generative AI in scientific writing

Generative AI and AI-assisted technologies should only be used in the writing process to improve the readability and language of the manuscript.

## Funding

This work was supported by the 10.13039/100014717National Natural Science Fund (Grant No. 82073277 and 82173247); Science and Technology Research Program of Chongqing Municipal Education Commission (Grant No. KJQN 202200456); Project of Chongqing Natural Science Foundation Innovation and Development Fund (Municipal Education Commission) (Grant No. CSTB2022NS CQ-LZX0023) and the Natural Science Fund of Chongqing (Grant No. CSTB2024NSCQ-MSX0282).

## Conflict of interests

The authors declare that they have no competing interests.
