# Supplementary material for: Intratumoral microenvironment remodeling by lncRNA ROLLCSC enhances lung adenocarcinoma progression
Source: Genes Dis. 2025 Aug 5;13(3):101788. doi: 10.1016/j.gendis.2025.101788 (PMC12914535; doi:10.1016/j.gendis.2025.101788)
Supplement: Multimedia component 1 [file mmc1.docx]

**
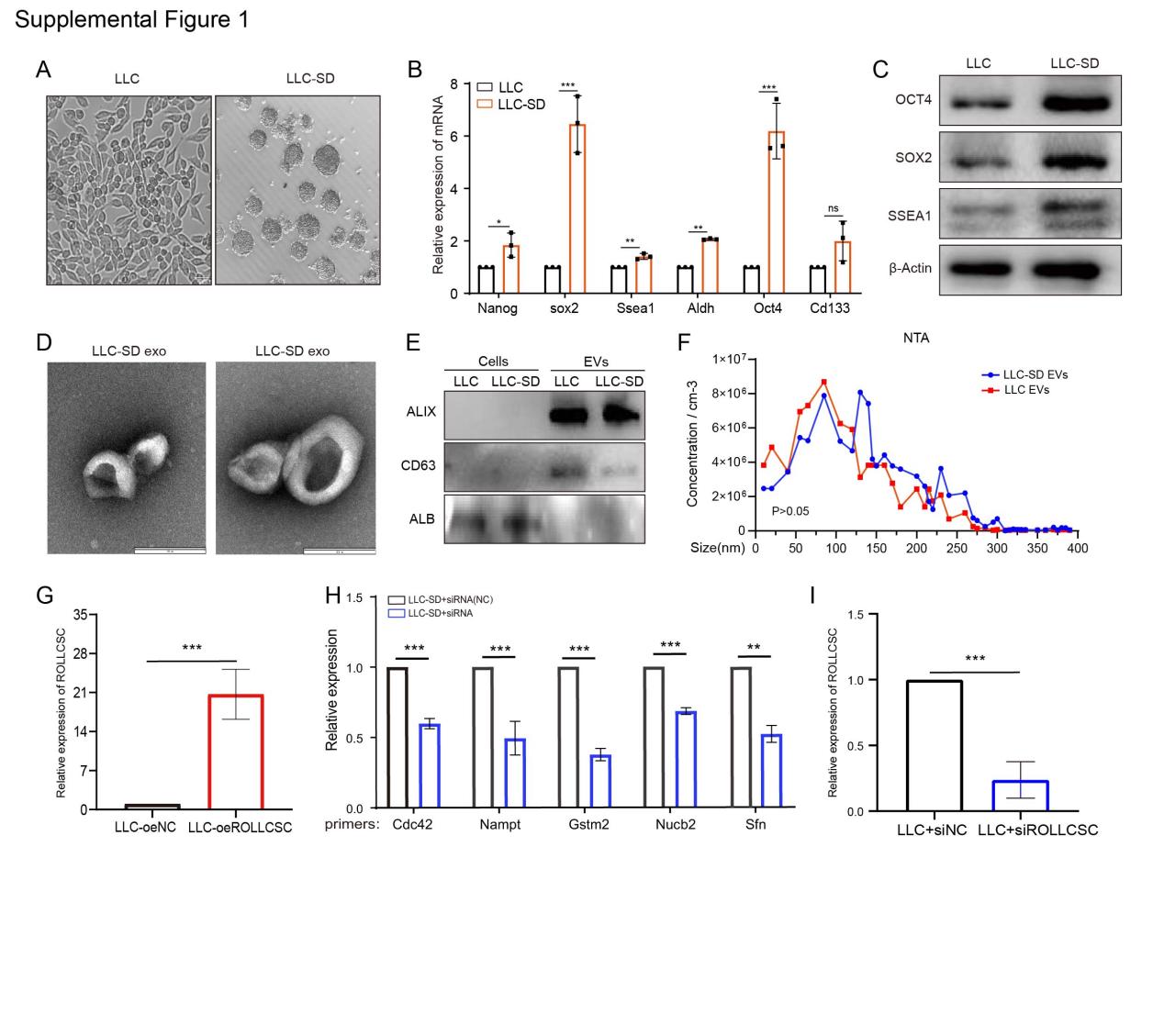
Supplemental Figure1：**

(A) Cell morphology observed under a microscope. (B) mRNA expression levels of stem cell markers in LLC-SD cells. (C) Protein expression levels of stem cell markers in LLC-SD cells.

(D) Morphology of extracellular vesicles observed under transmission electron microscopy. (e) Protein expression levels of extracellular vesicle markers in both cells and extracellular vesicles. (F) Nanoparticle Tracking Analysis analysis of extracellular vesicles. (G) PCR analysis of the overexpression efficiency of ROLLCSC lentivirus. (H) PCR analysis of siRNA knockdown efficiency in LLC-SD cells. (I) PCR analysis of siROLLCSC knockdown efficiency in LLC-SD cells.


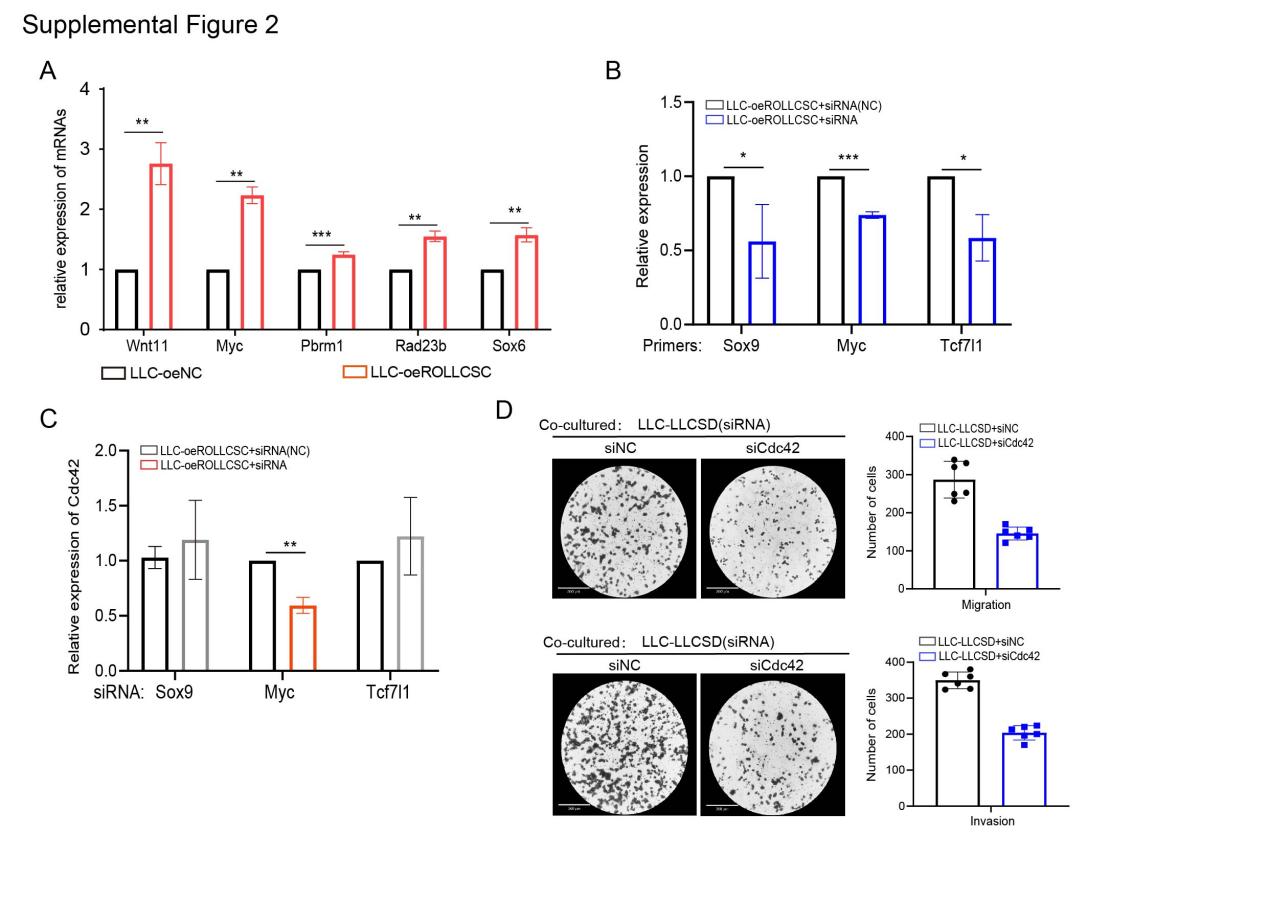


**Supplemental Figure2：**

(A) PCR analysis of the expression of Wnt/β-catenin pathway marker genes in LLC cells. (B) PCR analysis of siRNA knockdown efficiency in LLC-oeROLLCSC cells. (C) PCR analysis of *Cdc42* mRNA levels following transcription factor knockdown. (D) Representative images and statistical analysis of transwell assay results, showing the effect of *Cdc42* downregulation on the migration (right) and invasion (left) capabilities of LLC cells. Six randomly selected images per group were used for data analysis.


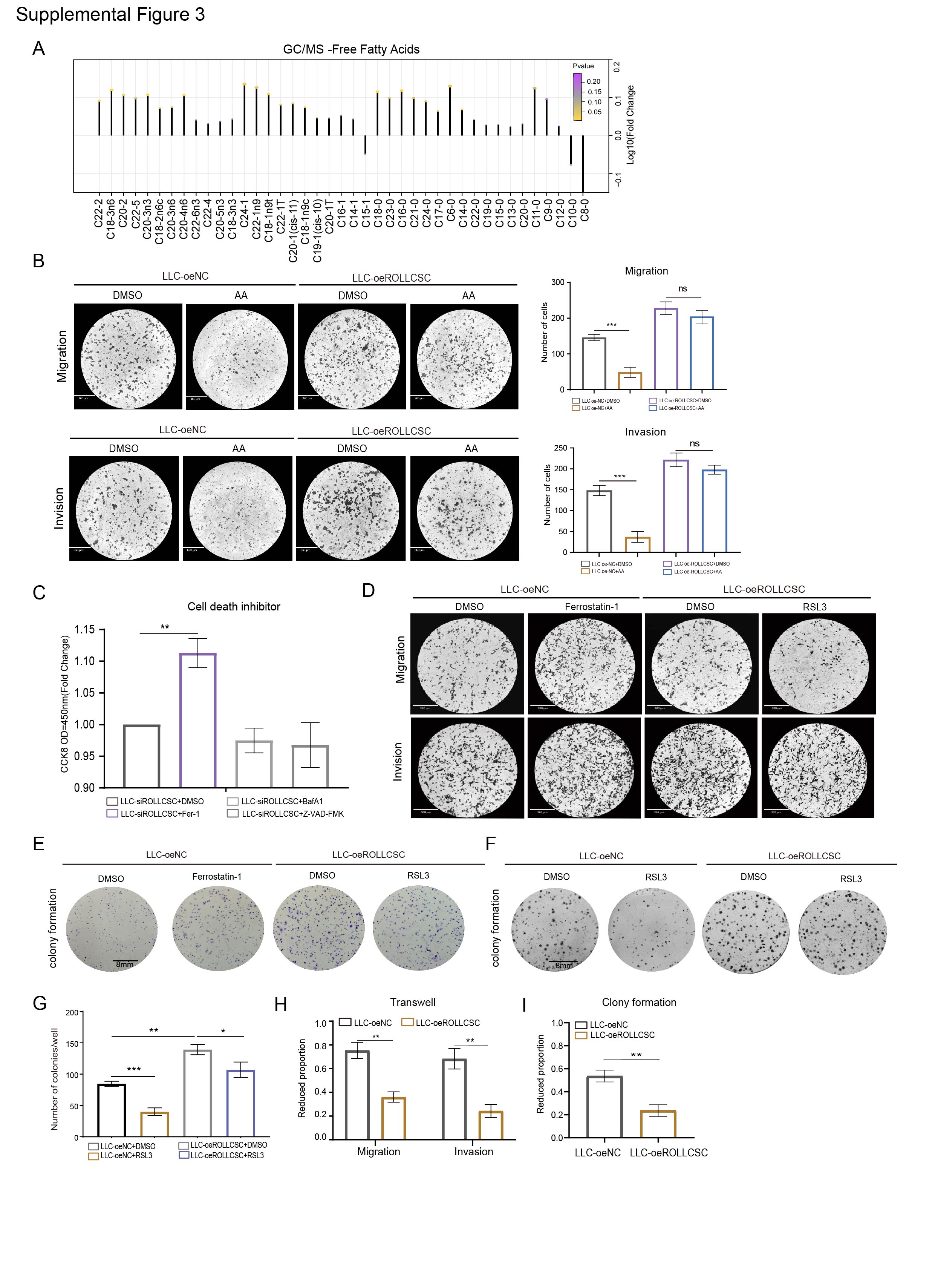


**Supplemental Figure3：**

1. Targeted free fatty acid GC/MS analysis was carried out in LLC cells, and the differential expression of free fatty acids in the overexpression ROLLCSC group was visualized, three biological replicates were performed in each group. (B) Representative images and statistical analysis of transwell assay results, Six randomly selected images per group were used for data analysis. (C) Regarding the effect of various cell death inhibitors on the viability of LLC-siROLLCSC cells, the absorbance value at 450nm was measured by a microplate reader after 2 hours of CCK-8 treatment. (D) Representative images of transwell assay results, Upper=migration, Down=invasion. (E)Representative images of colon formation results. (F-G) Representative images and statistical analysis of the results of the colony-forming assay show the effect of RSL3 on the colony-forming ability of LLC cells, n=3. (H) Proportion of decreased migration and invasion assays between different cells. (I) Proportion of decreased colony formation assays between different cells.


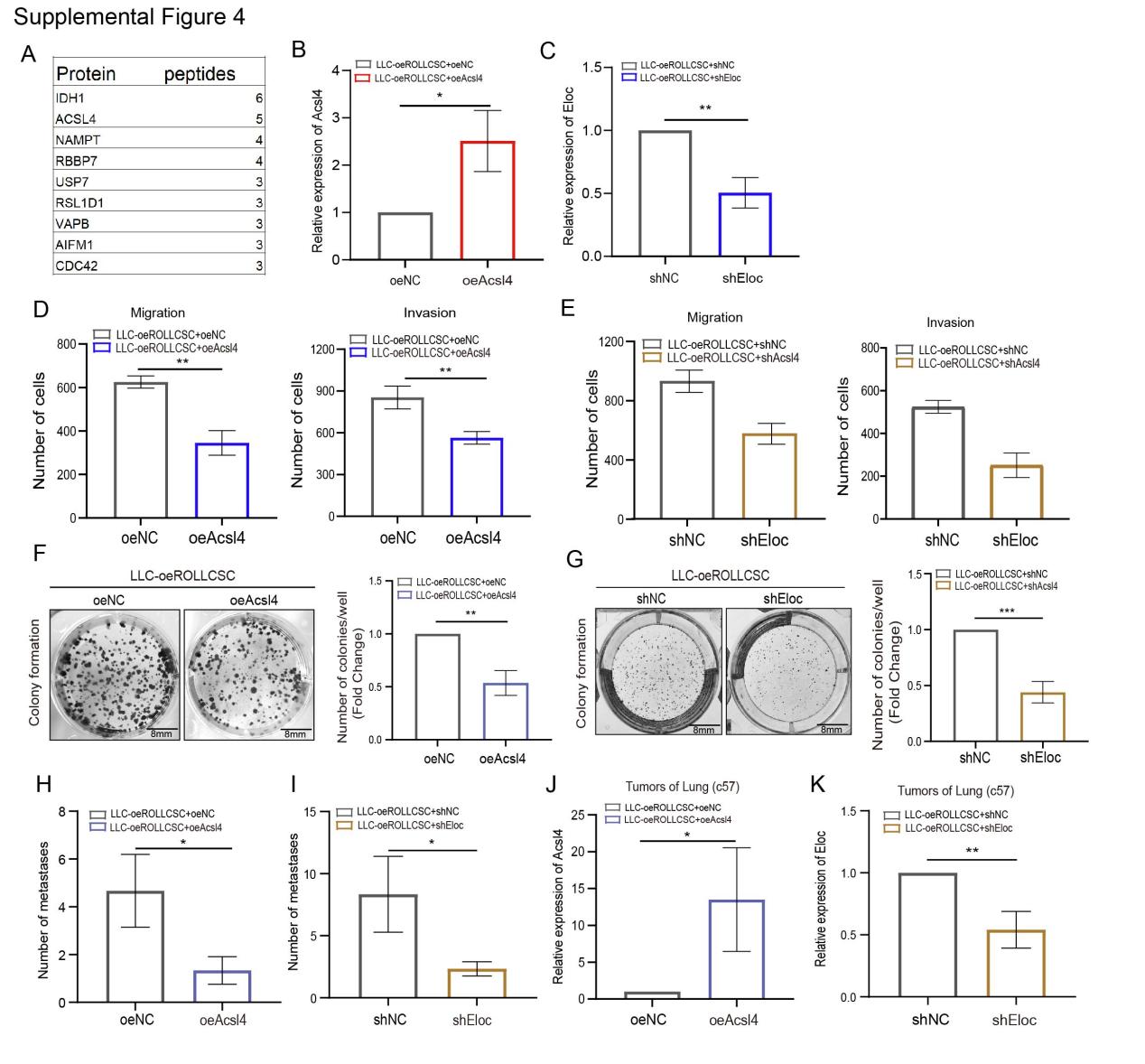


**Supplemental Figure4：**

(A) The proteins specifically bound to the sense-ROLLCSC were identified using mass spectrometry. (B) Transfection efficiency of *Acsl4* overexpression lentivirus. (C) Transfection efficiency of *Eloc* knockdown lentivirus. (D) statistical analysis of transwell results suggest the effect of *Acsl4* overexpression on the migration (left) and invasion (right) ability of LLC-oeROLLCSC cells, n=6. (E) statistical analysis of transwell results suggest the effect of Eloc knockdown on the migration (left) and invasion (right) ability of LLC-oeROLLCSC cells, n=6. (F) Representative images and statistical analysis of the results of the colony-forming assay show the effect of *Acsl4* overexpression on the colony-forming ability of LLC cells, n=3. (G) Representative images and statistical analysis of the results of the colony-forming assay show the effect of *Eloc* knockdown on the colony-forming ability of LLC cells, n=3. (H-I) Statistical results of lung metastases in mice in Fig. 5o-p experiments. (J) The mRNA expression of *Acsl4* in lung metastases of C57/BL6 mice was detected by PCR. (K) The mRNA expression of *Eloc* in lung metastases of C57/BL6 mice was detected by PCR.

**
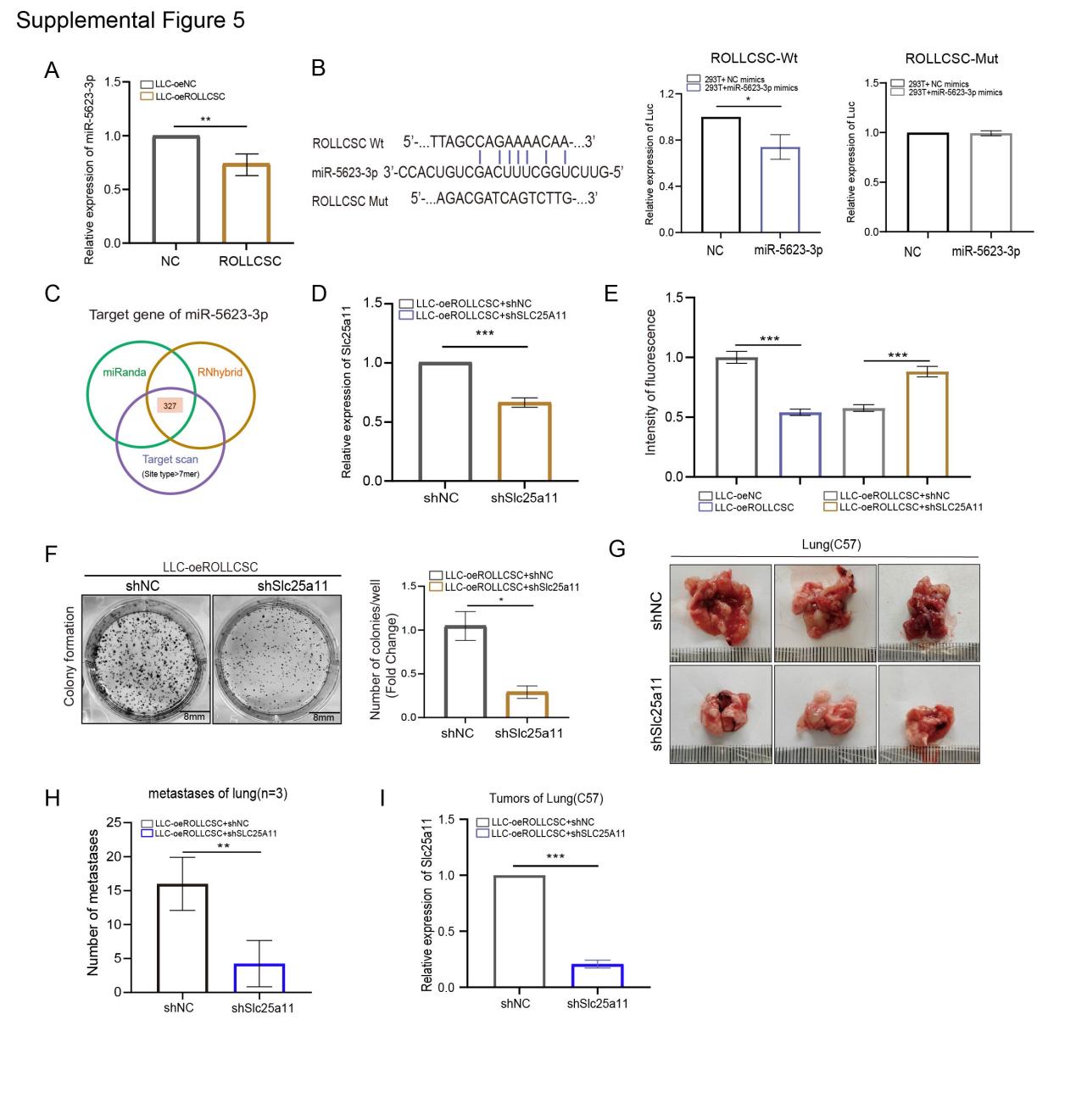
**

**Supplemental Figure5：**

(A) PCR analysis validating the regulatory effect of ROLLCSC on miR-5623-3p expression. (B) Dual-luciferase assay confirming the interaction between ROLLCSC and miR-5623-3p. (C) Venn diagram illustrating the target genes of miR-5623-3p identified through multiple databases. (D) Lentiviral transfection efficiency of *Slc25a11* knockdown. (e) Quantification of fluorescence in Fig. 6l using ImageJ. (F) Representative images and statistical analysis of the results of the colony-forming assay show the effect of *Slc25a11* knockdown on the colony-forming ability of LLC cells, n=3. (G) Lung tumor metastases in C57/BL6 mice under room light in Fig. 6o experiments. (H) Statistical results of lung metastases in mice. (I) The mRNA expression of *Acsl4* in lung metastases of C57/BL6 mice.
