# Supplementary material for: Intratumoral microenvironment remodeling by lncRNA ROLLCSC enhances lung adenocarcinoma progression
Source: Genes Dis. 2025 Aug 5;13(3):101788. doi: 10.1016/j.gendis.2025.101788 (PMC12914535; doi:10.1016/j.gendis.2025.101788)
Supplement: Multimedia component 2 [file mmc2.docx]

**LUAD patient samples**

All samples were obtained with written consent from participants and underwent rigorous ethical review. The balance of sample numbers and the reliability of diagnostic results for patients with lung adenocarcinoma were carefully maintained. Strict inclusion and exclusion criteria were adhered to, ensuring the integrity and validity of the study. The details are outlined below: Inclusion criteria:1) Patients with an accurate pathological diagnosis of primary lung adenocarcinoma; 2) Complete clinicopathological and follow-up data were available for each patient; 3) Surgical resection was the preferred treatment method; 4) Patients had signed the informed consent form, indicating their willingness to participate. The exclusion criteria are 1) Patients who died perioperatively; 2) Patients who received palliative surgery; 3) Patients who had undergone chemotherapy with any drugs. 4) Patients with a concurrent diagnosis of other types of cancer.

**RNA Pulldown Assay and Mass Spectrometry analysis**

The target RNA was prepared using a High Yield T7 Biotin16 RNA Labeling Kit (APExBio, K1082) in vitro transcription and labeled with biotin-UTP16 (APExBio, K1082). RNA obtained from in vitro transcription was incubated with streptavidin magnetic beads (APExBio, K1301) for 2 h at 4 ° C. Then, 1 x 10^7 cells were incubated with 1 ml Lysis buffer (APExBio, K1123) containing protease inhibitor (Beyotime, P1006) and RNase inhibitor (Beyotime, R0108) on ice for 10 minutes, followed by centrifugation at 12,000g for 10 minutes to collect the supernatant. The resulting lysate was then mixed with the above magnetic beads-RNA mixture and incubated overnight at 4℃. The following day, the beads were separated from the supernatant and washed thoroughly with 1xTBS buffer (Beyotime, ST661), and 50 μl SDS loading buffer (Solarbio, P1040) was added, boiled for 10 minutes, and the proteins were separated for subsequent MS analysis (APExBio).

**RNA Immunoprecipitation (RIP)**

The RIP assay was executed with precision using the RNA Immunoprecipitation Kit (Geneseed, P0102), the protocol was rigorously followed, including cell sample processing for RNA extraction and pre-treatment. Magnetic beads were prepared and conjugated with antibodies for 2-3 hours to ensure optimal antibody binding. Subsequently, an overnight antigen capture reaction was conducted to allow for efficient binding of RNA-protein complexes. After rigorous washing steps (5-10 washes) to remove unbound components, the RNA/protein complexes were eluted and purified. Further analysis was conducted using quantitative PCR (Q-PCR) and Western Blot methods to provide insights into the RNA-protein interactions.

**ChIP:**

ChIP assay was conducted using a ChIP kit (Beyotime, P2078, China). The antibodies utilized for ChIP are presented in Supplemental Tables. Primers were synthesized based on the CDC42 promoter region containing the Myc binding site (Supplementary Tables). The collected cells were sonicated into appropriate fragments and subsequently immunoprecipitated with Myc antibody at 4 °C. Eventually, the immunoprecipitated complexes were eluted with ChIP buffer, and the RNA was extracted and subjected to PCR reactions.

**Immunoprecipitation (IP) experiment**

Prepare about 1×10^7 cells, collect the cells and lysed with 1ml lysis buffer (APExBio, K1123), with 10μl protease inhibitors (Beyotime, P1006, China), lysed on ice or at 4℃ for 30 minutes, and then centrifuged to get the supernatant. After that, wash the A/G magnetic beads (APExBio, K1305) with an appropriate volume of 1x TBS. Then, add a suitable amount of antibody and incubate at 4℃ for 2 hours to allow the antibody to bind to the magnetic beads. After that, the supernatant of the cell lysate was added to the magnetic beads with the antibody, and incubated at 4℃ for 12 hours. Finally, centrifuge to remove the supernatant, leave the beads. Protein samples were obtained by washing the magnetic beads using 1x TBS (NCMbiotech, WB20500), adding 1x loading buffer (Solarbio, P1040)and boiling for 10 mins. Protein samples collected from this method are further examined by Western blotting or mass spectrometry to identify target proteins.

**Bioinformatic analyses and public database.**

To gain insights into the molecular mechanisms underlying the studied phenomena, bioinformatic analyses were conducted on publicly available datasets. Specifically, LUAD (Lung Adenocarcinoma) datasets were retrieved from TCGA, a comprehensive resource for cancer genomics data. The analysis of these datasets was facilitated by the use of dedicated tools accessible through the TCGA. To structure and preprocess the raw data, Perl (version 5.30.0.1) and R language (version 3.6.3) were employed. Perl scripts were utilized for data extraction and manipulation, while R language scripts facilitated the statistical and bioinformatic analyses. In particular, KEGG (Kyoto Encyclopedia of Genes and Genomes) , GO (Gene Ontology) enrichment analyses, and Gene Set Enrichment Analysis(4.2.3) were performed using the DAVID (Database for Annotation, Visualization, and Integrated Discovery) platform (ncifcrf.gov/david). The results of these enrichment analyses were then visualized using website (https://www.bioinformatics.com.cn/). Furthermore, survival analysis and prognosis analysis were also performed using GEPIA2.0, enabling the assessment of patient outcomes and the identification of prognostic factors. RNA library

**RNA library construction and RNA sequencing**

Ensure the integrity and purity of the extracted total RNA. Specifically, the ratio of 260/280 nm absorbance was maintained at 1.9-2.1, while the concentration was ensured to exceed 200 ng/μl. Subsequently, the VAHTS Universal V10 RNA-seq Library Prep Kit for Illumina (Vazyme, NR606-01), VAHTS mRNA Capture Beads (Vazyme, N401-01), and VAHTS RNA Adapters set for Illumina (Vazyme, N803-01) were utilized to generate the RNA sequencing library. The original data of RNA sequencing were obtained in fasta format by MGI. These data were then processed using the Mus_musculus.GRCm38 reference genome to perform gene matching and convert the data into an appropriate format for downstream analysis.

**ELISA Experiment**

The same number of cells were inoculated and the culture supernatant was collected. The change of AA in the cell supernatant was detected by The Mouse Arachidonic Acid ELISA KIT (Cusabio, CSB-E14102m) according to the instructions of the reagent, and then detected by microplate reader.

**Animal experiment for lung metastasis model**

The Animal Experiment Committee at Chongqing Medical University approved the conduct of the study, which adhered to strict ethical guidelines and regulations. Young female C57 mice, aged 6 to 8 weeks, were bred in a pathogen-free controlled environment. To establish a lung metastasis model, 5 × 10^5 tumor cells were injected into the left lung of each mouse in a volume of 30 μl. These mice were then observed every two days for five weeks. At the end of the observation period, the mice were euthanized humanely using cervical dislocation. Tumor metastases were then examined using a Sellstrom Z87 fluoroscope and histologically confirmed via Hematoxylin and Eosin (H&E) staining.

**H&E staining of tumor metastasis tissue**

Tumor-bearing tissue samples were excised from the mice and thoroughly washed three times with PBS. The tissues were then fixed in 4% paraformaldehyde for 72 hours at 4°C. Following fixation, the tissues were sent to the Basic Medical College of Chongqing Medical University for histological processing and Hematoxylin and Eosin (H&E) staining.

**Transwell assay**

The chambers and 24 well plates were purchased from Corning. 2 x 10^4- 5 x10^4 cells were added to the upper chamber and cultured in a serum-free environment. The lower chamber uses 20% FBS to form a concentration gradient. After 20-24 hours of culture, fixed with ice methanol for 30 minutes at 4 ℃, moistened with PBS, and stained with crystal violet (Beyotime, C0121) for 15 minutes. Migrated cells were viewed with Echo Laboratories RevolveFL (RVL-100-G, California), and processed using Image J 1.47 software

**Colony formation**

In a six-well plate, 500 cells were seeded per well using DMEM with 10% FBS medium. These cells were incubated in a controlled environment of 5% CO2 and 37°C for seven days. The cells were then fixed with 4% paraformaldehyde (Beyotime, P0090) and stained with crystal violet (Beyotime, C0121), and the number of cell spheres was counted, with spheres defined as clusters containing at least 50 cells.

**CCK-8 proliferation assay.**

CCK-8 cell counting kit (CA1210, Solarbio, China) determination for quantifying cell proliferation. 3000 cells were added to a 96-well plate and the growth was detected for 4 consecutive days. The microplate reader (Thermo, VARIOSKANLUX) was used to measure the absorbance of each hole at 450nm.

**Mitochondrial extraction**

The experiment was conducted utilizing the mitochondrial extraction kit (Solarbio, SM0020). For each extraction, 5×10^7 cells were necessary. The subsequent experimental steps were executed in strict accordance with the manufacturer's guidelines. Following the completion of the extraction process, the mitochondrial precipitates were resuspended in the Store Buffer (Solarbio, SM0020) and either used promptly or stored at -70℃ for future use.
