# Supplementary material for: Intratumoral microenvironment remodeling by lncRNA ROLLCSC enhances lung adenocarcinoma progression
Source: Genes Dis. 2025 Aug 5;13(3):101788. doi: 10.1016/j.gendis.2025.101788 (PMC12914535; doi:10.1016/j.gendis.2025.101788)
Supplement: Multimedia component 3 [file mmc3.docx]

#### Supplementary Tables:

**Table1: Primer sequence:**

| **Primer** | **Forward** | **Reverse** |
| --- | --- | --- |
| ROLLCSC | tgaacaaggacatccgaggc | ccctcctggtgtaccgagta |
| ROLLCSC sence | taatacgactcactatagggtggtcagatcgactgcaacg | caggcaagctgttcagcaaa |
| ROLLCSC antisence | tggtcagatcgactgcaacg | taatacgactcactatagggcaggcaagctgttcagcaaa |
| miR-5623-3p-primers | aagctgaggttctggctttcag |  |
| *Ptgs2* | ccctcctcacatccctgaga | actctgttgtgctcccgaag |
| *Gpx4* | gcaatgaggcaaaactgacg | ccaggattcgtaaaccacactc |
| *Slc25a11* | ttaccactgctgcttccatg | acgactttcagcagcacatc |
| SLC25A11 | ccgtcaagttcctgtttggg | agtgtaaatgcccctcaggc |
| *Gss* | agtgtatctcggagctgggt | cacacagggtaggggttgtc |
| *Tbp* | agcaacaacagcaggcagta | ggaagagttgtggggtctgg |
| *Myc* | agtgcattgacccctcagtg | gtgtctcctcatgcagcact |
| *Cdc42* | tcttgcttgttgggacccaa | ttgacagccttcagatcccg |

**Table2: Antibody**

| **Antibodies** | **Dilution** | **Brand** | **Cat No.** |
| --- | --- | --- | --- |
| ACSL4 | 1:5000 for WB； 4μg for IP | Proteintech | 66617-1-Ig |
| ELOC | 1:1000 for WB | Proteintech | 12450-1-AP |
| GPX4 | 1:1000 for WB | Proteintech | 67763-1-Ig |
| CDC42 | 1:1000 for WB | Proteintech | 10155-1-AP |
| Beta-catenin | 1:1000 for WB | cell signaling technology | 8480T |
| Phospho-beta-catenin | 1:1000 for WB | cell signaling technology | 4176T |
| SLC25A11 | 1:1000 for WB | Proteintech | 12253-1-AP |
| Ubiquitin | 1:1000 for WB | Proteintech | 10201-2-AP |
| β-actin | 1:10000 for WB | Proteintech | 81115-1-RR |
| IgG | 4μg for IP | Proteintech | 30000-0-AP |

**Table3: Dual luciferase plasmids**

| **Slc25a11-Wild type (Psicheck2):** | 5’-……….CTGGAGGACCTGGATTCTGCTACCCTGGGCTATTCTATTTATTTTCCCTCTCCAGTGTGGTTTTCTCCTTTGCGGTAAAGAACTTAGTCTGTTCTCCCCCTACTCCAGCCTGCCTGCTGTGCTTGCCTGATGCTTAGAATCACTTTGCCCCTGGCTCTTCCTGAGGAGACCTAAGACACACACCAAGGATTTCTAGTCTCCCTTGGGTGTTAGATTCAGGGCACACCAGACAGCAGATCCCTCTCTCTGGGGAATCCAAGGCAGAGCTGAGGAGACAGGAGAAAGCAGAAGCTGTCAAGATGACAAAGGGTCTATGGTGGAAGATGTGGCCTTCCTCCTCCCTGAGGACTTAATAAATTGGATTGAAATACCAACCCCAAATCTGGATAGTCTTAAGGATCATAGGGGGAGGTAGCTGCACAAAGAAGCTTATATCTCTGGAAAACAAACTGGAGTTTTAACAAAATAATTAAAGAAAGTCACCTACTG………-3’ |
| --- | --- |
| Slc25a11-Mutant type (Psicheck2): | 5’-………CTGGAGGACCTGGATTCTGCTACCCTGGGCTATTCTATTTATTTTCCCTCTCCAGTGTGGTTTTCTCCTTTGCGGTAAAGAACTTAGTCTGTTCTCCCCCTACTCCAGCCCTTACAACTATTTTGCCTGATGCTTAGAATCACTTTGCCCCTGGCTCTTCCTGAGGAGACCTAAGACACACACCAAGGATTTCTAGTCTCCTCTTGGGTGTTAGATTCAGGGCACACCAGACAGCAGATCCCTCTCTCTGGGGAATCCAAGGCAGAGCTGAAAGACTCTGAGAAAGCAGAAGCTGTCAAGATGACAAAGGGTCTATGGTGGAAGATGTGGCCTTCCTCCTCCCTGAGGACTTAATAAATTGGATTGAAATACCAACCCCAAATCTGGATAGTCTTAAGGATCATAGGGGGAGGTAGCTGCACAAAGAAGCTTATATCTCTGGAAAACAAACTGGAGTTTTAACAAAATAATTAAAGAAAGTCACCTACTG………-3’ |
| Cdc42 promoter region-Wlid type: | GATGGGGTAGATTAGGGTCAAGGAGTAACCCCACTCCAACTTGGGGTGGTGGAAAGGTCAAGGTCAAGGGTCACTTTCTGACAAAAAAAAAATCAGCCCAAGAGACAAAGTTAGTCCTTGTCCTCAGAATAGACGAGGAAAGGCCAAA**CACTTGG**GTGACATCCCATCAAGATACAGCAGCTGGTGGAAAGGCCAGACATTGTCTGGGACATTCATGGGACTTCGAGGCAGGATAAAAGAAAGGACACAGCCAACCTGCCTTGGGAACAGAGAAAACTGTACTTTCTCTCAAGCCAAAGAAATTGATGAGGTTTTTTTGTTTGTTTGTTTGTTTTGTTTTGTAATCACAAGTTGCCATTTATGGTCATCGGAGTGGAGGGTGTGGCTGAGTGGCAGAG**CACTTG**CCAAGTATCTGGTGAGCCCTGGGTCCTCCCTGGCATCGGAGAAAGGCTAGACAACTTCGATCCCTATAGGGTGAGATTGCCCACATAGGGAAATGCCCACAGGGTAGAATGCAGCCTTGCTATAAGCGGGGCTTCGTTCGACACAGAACGATGTTTACAGTACAGCTTTGAAAGAAGTAGATTGCAGGCTGGGAGGTGGCTTAGAGGGCAAAGTGATTGCTGCGCAGGTGTGAGGACTTGAGTTCGAATCCCTAGGGCCCACATAAAGCTGGATGCAGGAGCACACATCTGAGGTCTCAGAGTTCTGCCTCCTTAAGACACACCCTCCTGTTTTTTGAGAATGGATGTTTTGTATCCTAGGACACTCTGAATCTTCTATGCAACCAAGGGTCACCATAGATTTTTCTGCTTCCACCTCCCAAGTGCTGGGATTACAGCCATCCATCTCAGAGCTTGGATTATGAATGTGGTAGAGGCACTAACGTGGGGCTTCGTGCATTCTTGGCAAGCTCTCTACCGACTGGGCTACATCTCCAGCCCGCAAGA |
| Cdc42 promoter region-Mutant type: | GATGGGGTAGATTAGGGTCAAGGAGTAACCCCACTCCAACTTGGGGTGGTGGAAAGGTCAAGGTCAAGGGTCACTTTCTGACAAAAAAAAAATCAGCCCAAGAGACAAAGTTAGTCCTTGTCCTCAGAATAGACGAGGAAAGGCCAAAttgtcaaGTGACATCCCATCAAGATACAGCAGCTGGTGGAAAGGCCAGACATTGTCTGGGACATTCATGGGACTTCGAGGCAGGATAAAAGAAAGGACACAGCCAACCTGCCTTGGGAACAGAGAAAACTGTACTTTCTCTCAAGCCAAAGAAATTGATGAGGTTTTTTTGTTTGTTTGTTTGTTTTGTTTTGTAATCACAAGTTGCCATTTATGGTCATCGGAGTGGAGGGTGTGGCTGAGTGGCAGAGttgtcaaCAAGTATCTGGTGAGCCCTGGGTCCTCCCTGGCATCGGAGAAAGGCTAGACAACTTCGATCCCTATAGGGTGAGATTGCCCACATAGGGAAATGCCCACAGGGTAGAATGCAGCCTTGCTATAAGCGGGGCTTCGTTCGACACAGAACGATGTTTACAGTACAGCTTTGAAAGAAGTAGATTGCAGGCTGGGAGGTGGCTTAGAGGGCAAAGTGATTGCTGCGCAGGTGTGAGGACTTGAGTTCGAATCCCTAGGGCCCACATAAAGCTGGATGCAGGAGCACACATCTGAGGTCTCAGAGTTCTGCCTCCTTAAGACACACCCTCCTGTTTTTTGAGAATGGATGTTTTGTATCCTAGGACACTCTGAATCTTCTATGCAACCAAGGGTCACCATAGATTTTTCTGCTTCCACCTCCCAAGTGCTGGGATTACAGCCATCCATCTCAGAGCTTGGATTATGAATGTGGTAGAGGCACTAACGTGGGGCTTCGTGCATTCTTGGCAAGCTCTCTACCGACTGGGCTACATCTCCAGCCCGCAAGA |
| Myc-Wild type(pcDNA3.1): | CTGGATTTTTTTCGGGTAGTGGAAAACCAGCAGCCTCCCGCGACGATGCCCCTCAACGTTAGCTTCACCAACAGGAACTATGACCTCGACTACGACTCGGTGCAGCCGTATTTCTACTGCGACGAGGAGGAGAACTTCTACCAGCAGCAGCAGCAGAGCGAGCTGCAGCCCCCGGCGCCCAGCGAGGATATCTGGAAGAAATTCGAGCTGCTGCCCACCCCGCCCCTGTCCCCTAGCCGCCGCTCCGGGCTCTGCTCGCCCTCCTACGTTGCGGTCACACCCTTCTCCCTTCGGGGAGACAACGACGGCGGTGGCGGGAGCTTCTCCACGGCCGACCAGCTGGAGATGGTGACCGAGCTGCTGGGAGGAGACATGGTGAACCAGAGTTTCATCTGCGACCCGGACGACGAGACCTTCATCAAAAACATCATCATCCAGGACTGTATGTGGAGCGGCTTCTCGGCCGCCGCCAAGCTCGTCTCAGAGAAGCTGGCCTCCTACCAGGCTGCGCGCAAAGACAGCGGCAGCCCGAACCCCGCCCGCGGCCACAGCGTCTGCTCCACCTCCAGCTTGTACCTGCAGGATCTGAGCGCCGCCGCCTCAGAGTGCATCGACCCCTCGGTGGTCTTCCCCTACCCTCTCAACGACAGCAGCTCGCCCAAGTCCTGCGCCTCGCAAGACTCCAGCGCCTTCTCTCCGTCCTCGGATTCTCTGCTCTCCTCGACGGAGTCCTCCCCGCAGGGCAGCCCCGAGCCCCTGGTGCTCCATGAGGAGACACCGCCCACCACCAGCAGCGACTCTGAGGAGGAACAAGAAGATGAGGAAGAAATCGATGTTGTTTCTGTGGAAAAGAGGCAGGCTCCTGGCAAAAGGTCAGAGTCTGGATCACCTTCTGCTGGAGGCCACAGCAAACCTCCTCACAGCCCACTGGTCCTCAAGAGGTGCCACGTCTCCACACATCAGCACAACTACGCAGCGCCTCCCTCCACTCGGAAGGACTATCCTGCTGCCAAGAGGGTCAAGTTGGACAGTGTCAGAGTCCTGAGACAGATCAGCAACAACCGAAAATGCACCAGCCCCAGGTCCTCGGACACCGAGGAGAATGTCAAGAGGCGAACACACAACGTCTTGGAGCGCCAGAGGAGGAACGAGCTAAAACGGAGCTTTTTTGCCCTGCGTGACCAGATCCCGGAGTTGGAAAACAATGAAAAGGCCCCCAAGGTAGTTATCCTTAAAAAAGCCACAGCATACATCCTGTCCGTCCAAGCAGAGGAGCAAAAGCTCATTTCTGAAGAGGACTTGTTGCGGAAACGACGAGAACAGTTGAAACACAAACTTGAACAGCTACGGAACTCTTGTGCGTAA |
